# Acupuncture-related therapy for sudden sensorineural hearing loss: clinical efficacy and central mechanistic insights

**DOI:** 10.3389/fneur.2026.1861797

**Published:** 2026-08-06

**Authors:** Luqi Yuan, Weidong Shen, Wa Cai

**Affiliations:** Department of Acupuncture, Shanghai Shuguang Hospital Affiliated to Shanghai University of Traditional Chinese Medicine, Shanghai, China

**Keywords:** acupuncture, clinical efficacy, mechanism, narrative review, sudden sensorineural hearing loss

## Abstract

Sudden sensorineural hearing loss (SSNHL) is a common otolaryngological emergency, but its pathogenesis remains incompletely understood. Although acupuncture has recently become widely used as a complementary therapy for SSNHL in clinical practice, a comprehensive review of its central mechanisms is still lacking. Based on literature from 2020 to 2025 in databases such as PubMed, Web of Science, Embase, China National Knowledge Infrastructure (CNKI) and Wanfang Data (Wanfang), this narrative review synthesizes the latest research into the central mechanisms of acupuncture-related treatment for SSNHL, focusing on three key areas: auditory pathway plasticity, activation of the emotional regulation network, and functional integration of neurovascular units. Preliminary evidence suggests that acupuncture-related therapy may promote hearing recovery and alleviate associated symptoms via multiple target pathways. The proposed central mechanisms include modulation of functional connectivity in the temporal auditory cortex, regulation of limbic–subcortical emotional pathways, and enhancement of neurovascular coupling. This review provides a theoretical basis for a deeper understanding of the potential roles of acupuncture-related treatment in SSNHL, although further rigorous studies are needed to confirm these mechanistic links.

## 1 Introduction

Sudden sensorineural hearing loss (SSNHL) is defined as an unexplained hearing loss that occurs within 72 h, with a loss of ≥20 dB HL at least two adjacent frequencies (1). The incidence varies by country but generally shows an upward trend, peaking between the ages of 50 and 60. The incidence is equal between men and women (2–4). In addition to the primary symptom of SSNHL (hearing loss), patients often experience other symptoms, including vertigo, tinnitus, a sensation of fullness or pressure in the ear, abnormal sensations around the ear, hyperacusis or hyperresonance, and psychological symptoms. Given the global aging population, the number of people in the age group most susceptible to SSNHL is growing, and the number of cases is expected to increase steadily until 2050 (5). Furthermore, in recent years, the SSNHL patient population has shown a trend toward younger age groups (6). This change may be associated with factors such as shifts in modern lifestyles (7), infection with SARS-CoV-2 (8), activation of inflammatory responses, or changes in healthcare-seeking behavior (9).

**Figure 1:**
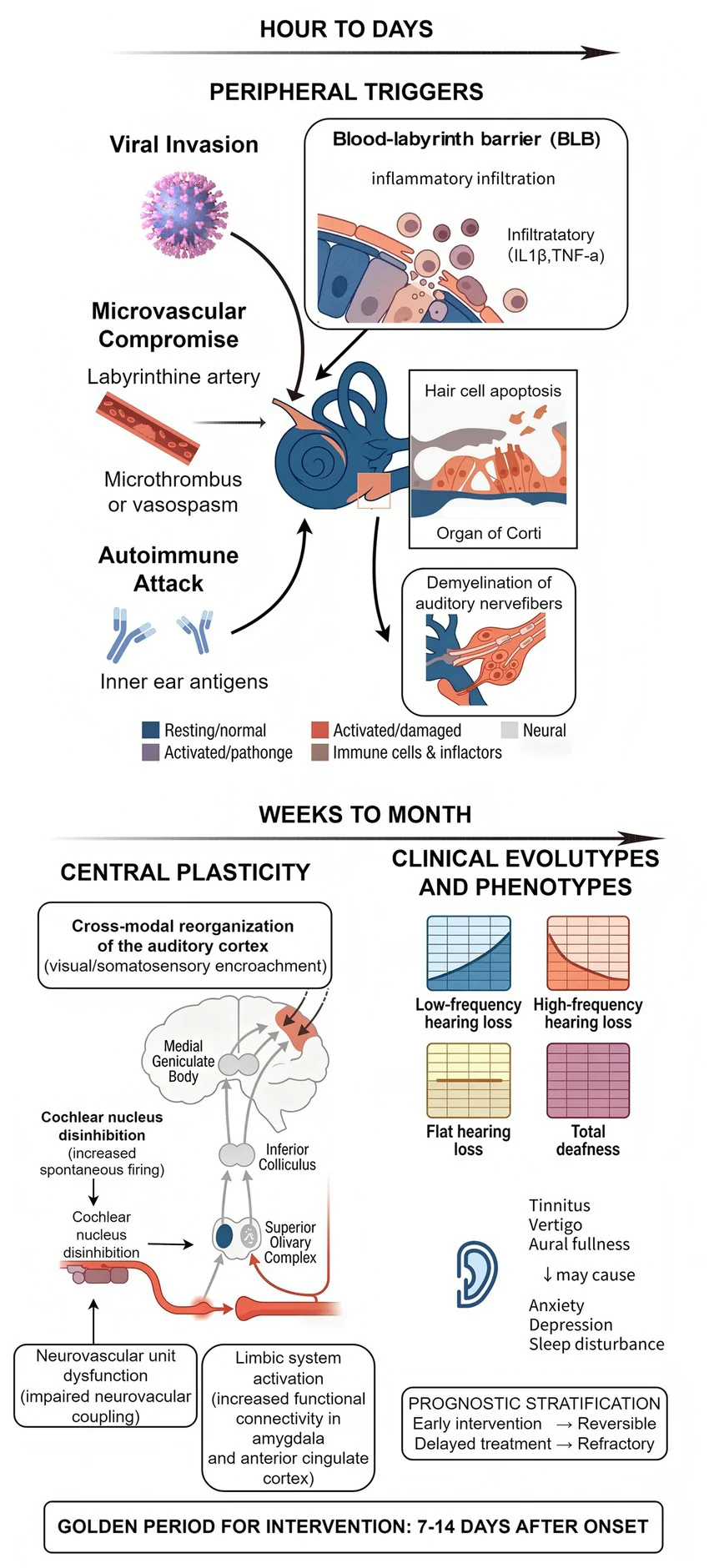
Schematic of the mechanism of sudden sensorineural hearing loss.

The pathophysiological mechanisms underlying SSNHL remain unclear, but may involve vascular, viral, autoimmune, infectious, and neoplastic processes (10) (Figure 1). Currently, there is no specific treatment for SSNHL. International guidelines support corticosteroids as a treatment option for SSNHL, with intratympanic steroid injection as salvage therapy, and suggest that hyperbaric oxygen therapy may be considered in combination with steroids within selected time windows (11). Chinese guidelines (1) additionally recommend agents that improve hemorheology as part of first-line or combination treatment. However, the routine use of vasodilators, vasoactive substances, or similar non-steroid pharmacologic treatments is not generally endorsed in major international guidelines. Systemic steroid therapy carries significant side effects for patients with underlying conditions such as hypertension, diabetes, and osteoporosis. Meanwhile, the efficacy of local administration—including intratympanic and retroauricular injections—is primarily reserved for alternative or salvage treatment. The efficacy of hyperbaric oxygen therapy remains controversial (12), with side effects including barotrauma of the middle ear and sinuses, as well as pulmonary and central nervous system oxygen toxicity (13).

The theoretical basis of acupuncture for hearing disorders can be traced to the *Huangdi Neijing* (The Yellow Emperor’s Classic of Internal Medicine), written over 2,000 years ago, which documented the pathological manifestations of hearing loss and proposed acupuncture strategies and acupoint selection protocols. According to traditional Chinese medicine theory, stimulating specific acupoints on the body corrects imbalances in Qi, Blood, Yin, and Yang, unblocks meridians, activates the body’s self-healing capacity, and restores hearing. From a biomedical perspective, it has been hypothesized that acupuncture may influence periauricular blood flow, hemorheology, and neural activity, although these mechanisms remain incompletely demonstrated in SSNHL.

Acupuncture is increasingly being used by Traditional Chinese Medicine practitioners as a complementary therapy for SSNHL. A previous clinical study (14) reported potential benefits of acupuncture in patients with refractory sudden deafness. Several meta-analyses have suggested that acupuncture, particularly when combined with Western medical comprehensive therapy, may be associated with improved outcomes in some studies (15, 16). However, the most recent of these meta-analyses also emphasizes that the included studies were generally of low methodological quality and at potential risk of bias, and that acupuncture monotherapy did not show a significant advantage over Western medical therapy in improving pure-tone hearing thresholds (15). The available evidence is therefore preliminary, mostly derived from studies with methodological limitations, and often based on combination therapies rather than acupuncture alone. Available reports suggest few adverse events, although safety reporting remains insufficient and does not allow firm conclusions regarding comparative safety.

Acupuncture-related therapies for SSNHL encompass a spectrum of techniques that can be broadly categorized into two groups. The first category includes traditional acupuncture modalities: manual acupuncture, in which fine filiform needles are inserted at specific acupoints and manipulated by hand to elicit the Deqi sensation; electroacupuncture, a modern adaptation that delivers a mild electric current via paired needles to enhance acupoint stimulation; and warm acupuncture, which combines needle insertion with the burning of moxa on the needle handle to provide both mechanical and thermal stimulation. The second category comprises combined and derivative approaches, where acupuncture is integrated with other strategies such as concurrent pharmacological agents, acupoint injection of bioactive substances, or auricular acupressure. In addition, transcutaneous electrical nerve stimulation (TENS) applied to acupoints has been investigated in some studies as a non-invasive alternative. Although TENS does not involve needle insertion, it shares with electroacupuncture the principle of delivering electrical current to acupoints and is therefore sometimes grouped under the broader category of acupoint stimulation therapies. While these combined protocols vary widely, they share the common principle of augmenting acupoint stimulation through multimodal intervention. This heterogeneity represents both a clinical strength and a challenge for evidence synthesis, as the specific contribution of each component is often difficult to isolate. In this review, we therefore distinguish between these approaches when summarizing clinical data.

Previous studies have primarily focused on assessing symptom severity before and after treatment, and there remains a need for more integrated, mechanism-oriented synthesis. This narrative review therefore aims to provide a comprehensive overview of recent clinical and preclinical evidence on acupuncture-related therapies for SSNHL, focusing on central mechanistic pathways, and to offer a theoretical reference for future clinical optimization and research.

## 2 Methods

### 2.1 Search strategy

Common clinical acupuncture methods used to treat sudden deafness include manual acupuncture, electroacupuncture, and warm acupuncture. This study conducted a structured search of PubMed, Web of Science, and Embase, using the keywords ‘acupuncture’, ‘electroacupuncture’, ‘warm acupuncture’, ‘transcutaneous electrical nerve stimulation (TENS)’, and ‘auricular acupressure’ in combination with ‘sudden deafness’, ‘sudden sensorineural hearing loss’, and ‘idiopathic SSNHL’. Relevant literature was sought in a comprehensive search of PubMed, Web of Science, Embase, China National Knowledge Infrastructure (CNKI), Wanfang Data Knowledge Service Platform, and VIP Chinese Journal Database (2020–2025). A small number of earlier studies were cited for background and mechanistic context (e.g., historical developments, foundational neurophysiological concepts) and were not part of the primary evidence synthesis. The search was conducted in English and Chinese databases without restrictions on document type or publication status. Consequently, only studies published in English or Chinese were included.

### 2.2 Literature screening

Clinical studies: All types of original clinical research investigating acupuncture-related interventions for SSNHL were considered, including randomized controlled trials, non-randomized controlled trials, and case series (*n* ≥ 5). To be eligible, studies were required to report at least one audiological outcome (e.g., pure-tone average change, hearing recovery rate) or validated symptom assessment (e.g., tinnitus severity, vertigo scales). Studies that combined acupuncture with pharmacotherapy or other interventions were included, provided the acupuncture component was clearly described.

Preclinical and mechanistic studies: Animal experiments and *in vitro* studies were included selectively when they provided direct evidence on the central mechanisms of acupuncture relevant to SSNHL—specifically, auditory pathway plasticity, limbic–cortical emotional regulation, or neurovascular unit function. These studies are discussed separately from clinical evidence (see Section 4) and are explicitly identified as preclinical in the text.

Exclusion criteria: The following publication types were excluded: reviews, meta-analyses, case reports (*n* < 5), expert opinions, commentaries, and conference abstracts. To minimize confounding from other etiologies, studies involving deafness secondary to clearly identified causes (e.g., trauma, ototoxicity, noise exposure, Meniere’s disease, acoustic neuroma) or severe systemic diseases were also excluded.

### 2.3 Data extraction

An initial screening of the titles and abstracts was independently conducted by two researchers. Following this initial phase, a secondary screening was performed via full-text review, and the results were subsequently cross-checked. Any discrepancies were resolved through joint discussion to reach a consensus. The methodological characteristics and key findings of the included clinical studies are summarized in Supplementary Table 1 (76–125).

## 3 Therapeutic effects on SSNHL

### 3.1 Audiometric outcomes

Hearing recovery is the primary indicator used to evaluate SSNHL treatment. Several clinical studies have suggested that acupuncture combined with conventional treatment may improve hearing outcomes, although the overall quality of evidence remains limited.

A retrospective observational study in Taiwan (17) compared acupuncture added to steroids and hyperbaric oxygen therapy versus steroids and hyperbaric oxygen alone in 154 patients with SSNHL. The proportion of patients with unsatisfactory hearing recovery was lower in the combined treatment group (16.22%) than in the control group receiving steroids and hyperbaric oxygen alone (44.19%, *p* = 0.008). Wang et al. (18) compared acupuncture alone with acupuncture combined with nasal-pinching Valsalva maneuvers in patients with early-stage SSNHL. The combined treatment group showed greater improvement in pure-tone hearing thresholds compared with the acupuncture-only control (27.00 ± 5.40 dB vs. 31.96 ± 7.09 dB, *p* < 0.05). In a study of 140 patients with SSNHL that compared two acupuncture modalities (19), the mean improvement in PTA was greater in the electroacupuncture group (−11.15 dB) than in the conventional acupuncture group (−6.32 dB). Tao et al. (20) found that when acupuncture was added to pharmacotherapy, the mean hearing threshold in the acupuncture-medication group (29.84 ± 11.01 dB) was lower than that in the Western medicine group (35.80 ± 13.13 dB, *p* < 0.05).

Shan et al. (21) compared electroacupuncture plus fire-needle therapy added to intratympanic prednisolone injection versus intratympanic injection alone in patients with refractory SSNHL. The combined treatment group showed a significantly higher overall response rate (52.4% vs. 29.3%, *p* < 0.05) and lower post-treatment PTA (69.28 ± 11.19 dB vs. 74.95 ± 10.31 dB, *p* < 0.05). Xu et al. (22) added warm acupuncture to a baseline regimen of oral prednisone acetate plus intravenous *Ginkgo biloba* extract in patients with SSNHL. The add-on group achieved a higher overall response rate compared with the pharmacotherapy-only control (97.30% vs. 78.38%, *p* < 0.05), with a between-group difference of approximately 19% points, and showed greater improvement in pure-tone audiometry at each treatment course (*p* < 0.05). The Linggui Eight Methods of acupuncture were evaluated in two studies with different control regimens. Zhang et al. (23) compared this approach with oral mecobalamin tablets combined with flunarizine hydrochloride tablets and reported a higher overall response rate (96.2% vs. 80.4%, *p* < 0.05) and lower post-treatment PTA (1.13 ± 0.12 dB vs. 1.92 ± 0.31 dB, *p* < 0.05) in the acupuncture group. Wang et al. (24) compared the same acupuncture approach added to intravenous methylprednisolone, batroxobin, and *Ginkgo biloba* extract versus the same pharmacotherapy alone in patients with flat-type SSNHL, and found a higher overall response rate (85.00% vs. 70.00%, *p* < 0.05) and lower post-treatment PTA (37.00 ± 7.13 dB vs. 41.35 ± 6.81 dB, *p* < 0.05), yielding a between-group difference of approximately 4.4 dB. Both studies suggested that the Linggui Eight Methods may be associated with better hearing outcomes for patients with different types of SSNHL.

Hu et al. (25) compared the addition of acupoint injection of dexamethasone plus mouse nerve growth factor to oral prednisone, *Ginkgo biloba* extract, and mecobalamin versus the same oral regimen alone in elderly patients with SSNHL. The add-on group achieved a higher overall response rate (91.07% vs. 75.44%, *p* < 0.05). Liao and Hua (26) compared intravenous dexamethasone, batroxobin, and *Ginkgo biloba* extract (Ginaton) alone versus the same regimen plus acupuncture in patients with unilateral SSNHL accompanied by vertigo. The acupuncture add-on group achieved a higher overall response rate (76.2% vs. 57.1%, *p* < 0.05), with a between-group difference of approximately 19% points. Luo et al. (27) compared acupoint injection of methylprednisolone sodium succinate at the Luxi (TE19) alone versus the same injection plus electroacupuncture in patients with unilateral SSNHL. The combination group achieved a higher overall response rate for hearing (95.0% vs. 83.3%, *p* < 0.05), with a between-group difference of approximately 12% points. Another study (28) compared electroacupuncture alone with electroacupuncture plus bloodletting therapy in patients with acute SSNHL. The combined treatment group achieved a higher overall response rate (89.7% vs. 64.1%, *p* < 0.05) and lower post-treatment PTA (42.08 ± 12.07 dB vs. 48.95 ± 12.85 dB, *p* < 0.05), yielding a between-group difference of approximately 6.9 dB.

### 3.2 Patient-reported symptoms

Several of the studies described above (19, 25–28) also assessed patient-reported symptoms, including tinnitus, vertigo, aural fullness, and emotional disturbances.

In the comparative study of electroacupuncture versus manual acupuncture (19), the reductions in Tinnitus Handicap Index (THI) and Dizziness Handicap Index (DHI) scores were more pronounced in the electroacupuncture group than in the conventional acupuncture group (both *p* < 0.01). In the add-on acupoint injection study by Hu et al. (25), higher efficacy rates for tinnitus (90.00% vs. 68.29%), ear fullness (88.24% vs. 69.70%), and vertigo (92.50% vs. 66.67%) were reported for the combined treatment group compared with the oral-medication-only control (*p* < 0.05). Liao and Hua (26) found that the acupuncture add-on group showed greater improvements in vestibular function, as reflected by lower oVEMP thresholds (86.0 ± 5.3 vs. 89.4 ± 5.4 dB nHL) and higher oVEMP response rates (81.0% vs. 52.4%) compared with the pharmacotherapy-only group (all *p* < 0.05). Similar improvements were observed for cVEMP parameters. Liu et al. (29) compared the addition of acupuncture at the sphenopalatine ganglion to vestibular rehabilitation training versus vestibular rehabilitation training alone in patients with unilateral SSNHL complicated by vertigo, with both groups receiving standardized pharmacotherapy. The add-on acupuncture group achieved a higher overall response rate for hearing (87.5% vs. 65.0%, *p* < 0.05) and greater improvements in DHI scores (total index: 12.41 ± 0.77 vs. 30.44 ± 1.97), BBS scores (50.73 ± 4.44 vs. 38.29 ± 5.94), and SAS scores (36.29 ± 4.59 vs. 44.17 ± 4.72) compared with the control group (all *p* < 0.05).

Fan et al. (30) evaluated a complex multi-modal TCM protocol: oral *Zhu Yu Tong Qiao Cong Er Decoction* (a traditional Chinese herbal formula with blood-activating, liver- and kidney-tonifying, and collateral-dredging properties) combined with acupoint injection of a multi-component mixture of corticosteroids, herbal extracts, vasoactive agents, and neurotrophic factors (detailed in the original study). The herbal add-on group achieved a higher overall response rate (91.18% vs. 70.59%, *p* < 0.05), with a between-group difference of approximately 21% points, and showed shorter time to resolution of ear fullness (7.00 ± 3.15 d vs. 8.79 ± 2.10 d), tinnitus (7.55 ± 2.68 d vs. 9.41 ± 3.22 d), and vertigo (10.03 ± 3.25 d vs. 11.50 ± 2.00 d) compared with the control group (all *p* < 0.05). Jiang et al. (31) reported that accompanying symptoms resolved alongside hearing recovery after combined acupuncture and Long’s Orthopedic Manipulation. Sun et al. (32) compared conventional medication (vasodilators, corticosteroids, and neurotrophic agents) alone versus the same medication plus acupuncture in patients with sudden deafness and found lower scores for deafness, tinnitus, vertigo, nausea and vomiting, and ear fullness in the acupuncture add-on group (*p* < 0.05).

Regarding emotional symptoms, Zhang et al. (23) reported that adding acupuncture to conventional treatment (glucocorticoids, neurotrophic agents, and *Ginkgo biloba* extract) was associated with lower Self-Rating Anxiety Scale (SAS) and Self-Rating Depression Scale (SDS) scores in patients with SSNHL. Wang et al. (33) compared conventional nursing care combined with mindfulness training alone versus the same regimen with additional auricular acupressure in patients with SSNHL. After 10 days, the auricular acupressure add-on group showed lower scores on the SAS, SDS, and Pittsburgh Sleep Quality Index (PSQI) (*p* < 0.05), and shorter time to symptom resolution for hearing loss (10.12 ± 1.33 d vs. 10.83 ± 2.01 d), tinnitus and vertigo (8.36 ± 1.25 d vs. 9.17 ± 2.12 d), ear fullness (10.32 ± 1.09 d vs. 11.03 ± 2.14 d), and nausea and vomiting (8.68 ± 1.34 d vs. 9.35 ± 1.51 d) (all *p* < 0.05).

The clinical evidence reviewed here, while still preliminary, suggests that acupuncture-based interventions may contribute to improved hearing and alleviation of accompanying symptoms such as tinnitus, vertigo, and ear fullness. These interventions were most frequently evaluated as an add-on to standard pharmacotherapy. However, because most studies assessed combination protocols, the specific contribution of acupuncture per se cannot be reliably isolated. Moreover, not all studies have reported positive findings; several have observed no significant advantage of adding acupuncture to corticosteroid-based therapy or conventional Western medicine, as detailed in Section 5. There is also some indication that these interventions could help relieve anxiety and depression, though these findings require confirmation in larger, well-controlled trials. These clinical observations provide a rationale for discussing possible central mechanisms, although direct evidence linking clinical improvement to central nervous system reorganization remains limited.

## 4 Mechanisms of acupuncture-related treatment for SSNHL

**Figure 2:**
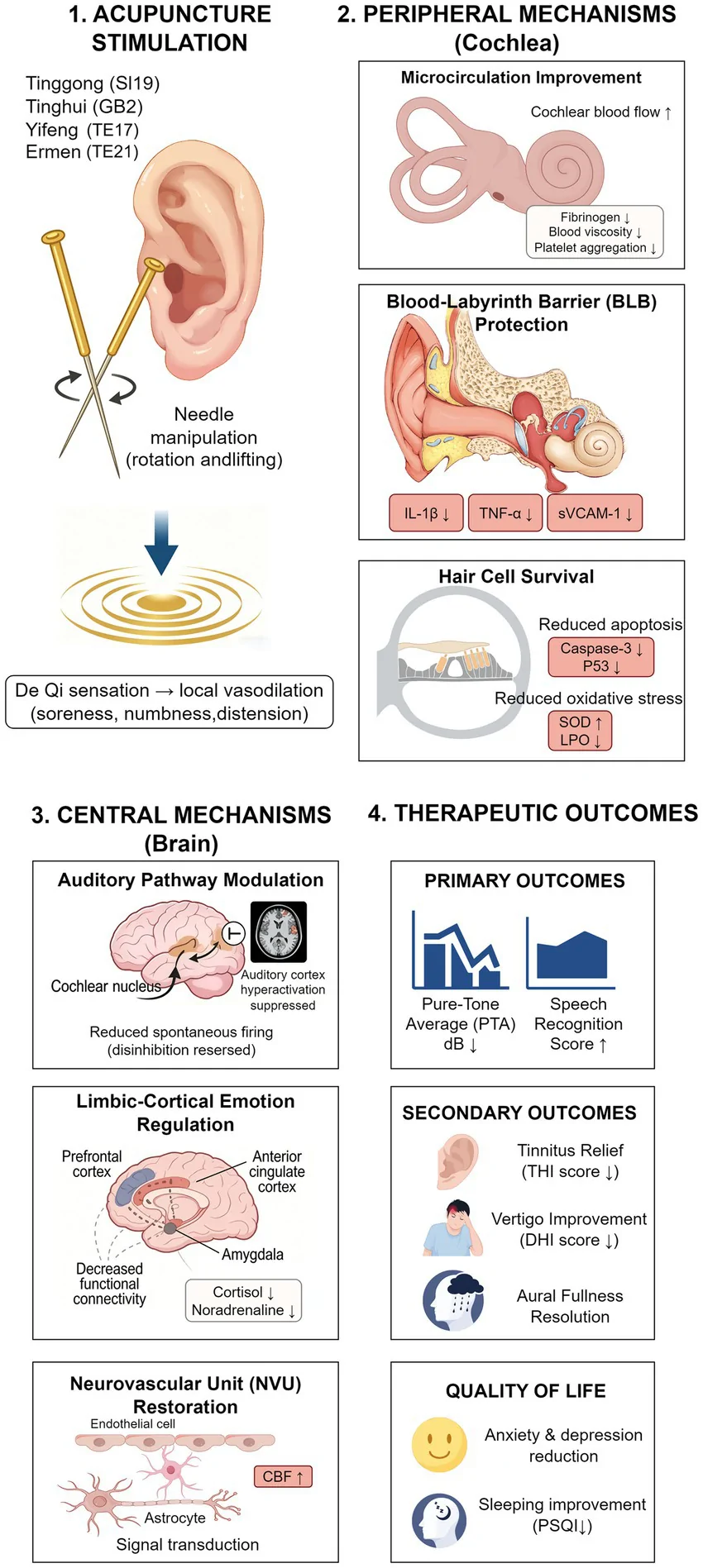
Mechanism diagram of acupuncture treatment for sudden hearing loss.

As introduced in Section 1, the pathophysiology of SSNHL involves vascular, viral, and immune-inflammatory mechanisms. Acupuncture, as a neuromodulatory intervention, has been proposed to influence these central changes through multiple potential pathways. The proposed mechanisms discussed in the literature include the following three aspects: modulation of auditory pathway plasticity, regulation of the limbic–cortical emotional network, and functional integration of neurovascular units (Figure 2). The following sections examine how acupuncture may intervene in these processes at the central level.

### 4.1 Modulation of auditory pathway plasticity

Damage to the hair cells in the inner ear or to the auditory nerve pathway leads to a sudden reduction in the signals received by the auditory nerve from the inner ear. This abrupt reduction in input signals from the affected side decreases the excitatory input received by neurons in the cochlear nucleus on the same side. Concurrently, a reduction in inhibitory input triggers a disinhibition phenomenon, leading to an abnormal increase in the spontaneous firing rate of neurons. This abnormal neural activity propagates through the auditory pathway and may be misinterpreted by the central nervous system as a real auditory signal, thereby generating tinnitus (34). The auditory brainstem plays a critical role in this process, as accumulating evidence indicates that brainstem nuclei serve not merely as passive relay stations but as active sites of neural remodeling that contribute to tinnitus generation and maintenance (35). This mechanism is considered one of the early central origins of tinnitus secondary to SSNHL, involving processes of reorganization and hypersensitization in the auditory cortex. It should be noted that tinnitus in general, tinnitus associated with chronic hearing loss, and tinnitus specifically triggered by acute SSNHL may involve overlapping but not identical neurophysiological substrates. In SSNHL, the suddenness of auditory deprivation may produce a distinct pattern of maladaptive plasticity compared with the gradual compensation observed in progressive hearing loss. Beyond its auditory components, tinnitus carries a substantial neuropsychological burden, with studies documenting significant associations between tinnitus severity and emotional distress, cognitive complaints, and reduced quality of life, particularly in older populations (36). These neuropsychological dimensions are especially relevant in SSNHL, where tinnitus often coexists with acute emotional stress and may exacerbate anxiety and depression.

Following unilateral hearing loss, morphological or functional changes occur in the auditory cortex and other sensory cortical areas, specifically manifested by the brain regions responsible for that sense being recruited by other intact senses. This phenomenon is known as cross-modal reorganization (37), meaning that the auditory cortex is appropriated by other intact sensory modalities. In patients with bilateral deafness, task-related functional magnetic resonance imaging (fMRI) scans show increased activity in auditory-related brain regions when performing visual or tactile tasks. Studies on preschool children with sensorineural hearing loss (38) show that children with hearing impairments exhibit new brain functional compensation phenomena, and adaptive reorganization occurs in the corresponding brain regions. Their language recovery may be associated with these regions undergoing functional compensation and reorganization. These findings demonstrate that the auditory cortex retains the capacity for cross-modal reorganization under conditions of profound or congenital auditory deprivation. However, whether acute unilateral SSNHL produces the same type of maladaptive cross-modal reorganization, and to what extent such reorganization competes with native auditory recovery, remains to be directly demonstrated in SSNHL-specific neuroimaging studies. It has been hypothesized that, by providing patterned somatosensory and neuromodulatory input, acupuncture could potentially help maintain auditory cortical responsiveness and create more favorable conditions for hearing recovery. However, this hypothesis has not been tested with direct neuroimaging evidence in SSNHL patients, and the proposed link between acupuncture-induced cortical modulation and clinical improvement remains speculative at present.

Earlier intervention is a well-known prognostic factor in SSNHL independent of the treatment modality, and may reflect the natural history of the disease, spontaneous recovery patterns, baseline severity, and treatment delay. Shi et al. (10) compared patients who received acupuncture within 14 days of SSNHL onset with those who received acupuncture after 14 days. The early intervention group achieved a higher overall response rate (80.00% vs. 53.33%, *p* < 0.05) and lower THI scores both post-treatment and at follow-up (*p* < 0.05). While these findings are consistent with a possible time-dependent effect, they do not isolate a specific acupuncture-related neuroplastic window, as the observed advantage of early treatment may partly reflect differences in spontaneous recovery potential and baseline characteristics between early- and late-treated groups. It remains plausible that delivering acupuncture during the acute phase, when the central auditory pathway may be more responsive to neuromodulatory input, could create more favorable conditions for recovery, but this hypothesis requires confirmation through controlled studies that directly compare early and late acupuncture while accounting for these confounding factors.

Acupuncture-related interventions have been hypothesized to systematically modulate abnormal plasticity in the aforementioned auditory pathways. At the brainstem level, studies of the auditory brainstem response (ABR) provide suggestive electrophysiological evidence that acupuncture-related interventions may modulate auditory pathway function. In patients with SSNHL, Gong et al. (39) compared ABR findings before and after treatment in acupuncture and sham acupuncture groups. They found that acupuncture at the bilateral Fengchi (GB20) and Gongxue (Extra)—a point located approximately 40 mm below GB20 in the posterior cervical region, first described by Gao Weibin (40, 41)—resulted in a more significant improvement in the inter-peak latency of ABR peaks I-V than sham acupuncture, consistent with improved conduction function from the auditory nerve to the brainstem. Chen et al. (42) conducted a comparative analysis of auditory brainstem evoked potential test results and found that electroacupuncture, as well as electroacupuncture combined with herbal injection at acupoints, can enhance the excitability and conduction of the cochlea, auditory nerve, and lateral collicular pontine regions in patients. In an animal study using healthy Sprague–Dawley rats, electroacupuncture at the right Zhongzhu (TE3) acupoint altered the characteristics of auditory evoked potentials (43). The latencies of waves I, III, and V, as well as the inter-peak latencies between I-III and I-V, were prolonged at specific time points following intervention. Among the long-latency responses indicative of cortical processing, the latency of the early cortical component (P0 wave) also underwent significant changes. These preclinical findings from healthy animals support the biological plausibility that electroacupuncture may modulate auditory signal conduction and cortical processing, but they cannot be directly extrapolated to the acute pathological state of SSNHL in humans, and they do not constitute evidence of clinical efficacy or disease-specific central mechanisms.

In a preclinical study (44), acupuncture using the “Strengthening the Spleen and Nourishing the Kidney” method was administered to C57BL/6 J mice, a strain that exhibits accelerated age-related hearing loss (45, 46). The ABR thresholds at the 12 kHz frequency level in the acupuncture group were lower than those in the control group. Although this model does not replicate the acute vascular or inflammatory events characteristic of SSNHL, it provides preclinical evidence supporting the hypothesis that acupuncture could potentially help preserve peripheral auditory function under conditions of ongoing cochlear degeneration—an effect that may involve improved cochlear microcirculation and reduced oxidative stress, mechanisms relevant to the neurovascular unit dysfunction discussed in Section 4.3. However, whether similar neuroprotective effects occur in SSNHL patients has not been directly demonstrated.

### 4.2 Regulation of the limbic–cortical emotional network

Stress disorder theory suggests that when the body is exposed to high-intensity stressors such as physical fatigue, disrupted dietary rhythms, significant emotional fluctuations, negative life events, insomnia, and anxiety, it can trigger dysfunction in the microcirculation, immune, and neuroendocrine systems. These pathophysiological changes may lead to the onset of SSNHL, which is also a potent stressor. Previous studies (47) have shown that hearing loss is closely associated with emotional disorders such as anxiety and depression, as well as sleep disorders. There may also be complex bidirectional relationships among these conditions. It has been reported that anxiety and depression scores in SSNHL patients are higher than in healthy individuals, and that changes in their brain functional gradients occur. Specifically, the functional gradient value in the cortex surrounding the right calcarine fissure is negatively correlated with anxiety scores (48). Adverse psychological states can further activate the sympathetic-adrenal axis, prompting the massive release of catecholamines, which in turn leads to changes in inner ear vascular and neural function, exacerbates inner ear vasospasm, and creates a vicious cycle (49, 50). Based on temporal bone pathology, Merchant et al. (51) proposed that SSNHL may result from the pathological activation of cellular stress pathways such as the nuclear factor kappa B (NF-κB) signaling pathway. Therefore, regulating activity in emotion-related brain regions to break the ‘hearing loss-emotional disturbance’ vicious cycle is a key therapeutic target for SSNHL.

Clinical studies have reported that acupuncture-based interventions may be associated with improved emotional state in some patients with SSNHL. Sun et al. (52) used a combination of acupuncture and psychological intervention to treat SSNHL and reported improvements in haemorheological parameters along with stable levels of depression and anxiety in the treatment group. Ma et al. (53) combined acupuncture with puerarin and hyperbaric oxygen therapy and observed greater improvement in stress markers compared with the control group. Although these results are preliminary, they suggest that acupuncture may help modulate the stress and emotional responses associated with SSNHL.

The central mechanisms underlying emotional regulation involve the limbic-paralimbic network, including the amygdala, hippocampus, anterior cingulate cortex, and insula. Studies using resting-state fMRI (54) revealed that, among the eight subregions of the amygdala, the bilateral basolateral subregion, the left striatal subregion, and the right superficial subregion exhibit abnormal functional connectivity with the whole brain. These regions serve as key nodes in emotional dysregulation. Animal studies have further elucidated the molecular mechanisms by which acupuncture modulates emotional pathways. Zhang et al. (55) applied electroacupuncture to the auricular region in mice with acute stress disorder. They found that the immobility time was reduced, an increase in the number of tyrosine hydroxylase (TH)-positive neurons in the ventral tegmental area (VTA). There was also upregulation of dopamine D1 receptor (DRD1) expression and downregulation of D2 receptor (DRD2) expression, suggesting that electroacupuncture may improve depression-like behaviors by regulating dopaminergic pathways in the mesolimbic system. The clinical studies reviewed above suggest that acupuncture-based interventions may be associated with reduced anxiety and depression levels in some patients with SSNHL. However, these studies involved combined interventions—such as acupuncture plus psychological intervention or acupuncture plus medication and hyperbaric oxygen therapy—making it difficult to isolate the specific contribution of acupuncture to the observed emotional improvements. The central mechanisms underlying any potential effect of acupuncture on emotional regulation in SSNHL have not been directly demonstrated in SSNHL populations. Studies on related conditions, including depression, presbycusis with tinnitus, and acute stress disorder in animal models, provide useful contextual evidence but do not establish that acupuncture modulates the limbic–cortical emotional network specifically in SSNHL. It remains an indirect hypothesis, extrapolated from these findings, that similar pathways may contribute to improved emotional outcomes in SSNHL. Direct neuroimaging studies in SSNHL patients receiving acupuncture are needed to evaluate this hypothesis.

### 4.3 Functional integration of the neurovascular unit

The neurovascular unit (NVU) is a dynamic functional complex composed of neurons, astrocytes, microglia, vascular endothelial cells, pericytes, and the basement membrane. Its primary function is to achieve precise matching between neural activity and local cerebral blood flow (CBF), known as neurovascular coupling (NVC) (56). Recent studies have revealed widespread abnormalities in neurovascular coupling function among patients with SSNHL, and these changes are closely associated with levels of inflammatory factors (57). One study (58) conducted a comparative analysis of NVC changes between SSNHL patients and healthy controls, examining their relationship with blood inflammatory factor levels. The results showed that global neurovascular coupling was reduced in the patient group, primarily affecting the auditory center. NVC indices showed significant correlations with inflammatory markers, suggesting that inflammation may contribute to the pathophysiological mechanisms of SSNHL by affecting the function of neurovascular units.

The integrity of neurovascular units depends on the structural and functional stability of the blood–brain barrier (BBB). Systemic inflammation can stimulate endothelial cells to express adhesion molecules, disrupting tight junctions between cells and facilitating the infiltration of inflammatory factors into tissues of the central nervous system (57). Chronic inflammation can also lead to endothelial dysfunction, causing the blood of patients with SSNHL to remain in a hypercoagulable state, which increases the risk of thrombus formation and results in impaired inner ear microcirculation (59). Disruption of inner ear microcirculation is a central component of the peripheral pathology in SSNHL (60), but dysfunction of the central neurovascular unit also needs to be considered. Animal studies have shown that specific restoration of vascular endothelial cell signaling pathways is sufficient to prevent hearing loss in NDP knockout mice (61), suggesting that vascular endothelial function is crucial for maintaining the auditory system. A hypercoagulable state induced by fibrinogen can lead directly to reduced cochlear blood flow and hearing loss in guinea pigs, whereas defibrinogen treatment can restore cochlear microcirculation and hearing (62, 63). Kanzaki et al. (64) found that elevated serum fibrinogen levels within 7 days of onset are associated with poor prognosis in patients with SSNHL. These studies suggest that improving vascular function may be a key mechanism underlying the therapeutic effects of acupuncture.

Some clinical studies have reported changes in systemic vascular, endothelial, hemorheological, or microcirculatory markers after acupuncture-related interventions; however, direct evidence that acupuncture restores central neurovascular coupling in SSNHL is lacking. As indirect evidence, Chang et al. (65) reported that a combination of acupuncture and *Longdan Xiegan Decoction* (a traditional herbal formula detailed in the original study) improved microcirculatory parameters—including increased serum nitric oxide levels and decreased endothelin and soluble vascular cell adhesion molecule-1 levels—in elderly patients with senile sensorineural hearing loss. Although this study did not enroll SSNHL patients, its findings provide preliminary support for the hypothesis that acupuncture-related interventions may have favorable effects on vascular endothelial function in sensorineural hearing loss more broadly. Zhou et al. (66) treated SSNHL with auricular acupuncture combined with acupoint application, resulting in improved hearing and significant reductions in high- and low-shear whole blood viscosity, as well as an increase in erythrocyte deformability index and improvement in nailfold microcirculation scores. These findings suggest that acupuncture-related interventions may be associated with improvements in systemic microcirculatory markers. Li et al. (67)’s results showed significant improvements in hemorheological parameters in the acupuncture group, along with reduced serum P53 protein and caspase-3 levels, suggesting that acupuncture may exert a protective effect by inhibiting cochlear cell apoptosis and improving inner ear microcirculation. Although these studies report favorable changes in systemic vascular, endothelial, and hemorheological parameters, they do not directly measure neurovascular coupling within the central auditory pathway. It remains plausible that acupuncture could contribute to restoring neurovascular unit function, but this hypothesis will require confirmation through studies that combine acupuncture intervention with direct measures of central neurovascular coupling, such as functional near-infrared spectroscopy or arterial spin labeling MRI, in SSNHL patients.

## 5 Null and neutral findings

Beyond these methodological considerations, the literature summarized in this review is not uniformly positive, and several studies have reported null or neutral results that warrant acknowledgement. A recent large-sample meta-analysis of 28 randomized controlled trials involving 2,456 patients found that, although acupuncture combined with Western medical comprehensive therapy (WMCT) was associated with a higher total effective rate and greater hearing threshold improvement compared with WMCT alone, there was no statistically significant difference between acupuncture monotherapy and WMCT in improving pure-tone hearing thresholds (MD = −5.45 dB, 95% CI: −20.75–9.85, *p* = 0.48) (15). In addition, individual clinical studies have yielded mixed findings. A 2025 retrospective study of 248 patients (68) found that adding batroxobin and acupuncture to corticosteroid therapy was not associated with greater hearing improvement than corticosteroid therapy alone (*p* > 0.05). Similarly, a 2001 clinical observation (69) reported that the combination of electroacupuncture and acupoint injection with Compound Danshen Injection achieved an overall response rate of 76.1%, compared to 78.8% in the conventional Western medicine group (*p* > 0.05). These divergent findings likely reflect differences in treatment protocols, patient characteristics, outcome measurement methods, and follow-up durations, and they caution against overgeneralizing the efficacy of acupuncture for SSNHL.

## 6 Limitations

Several limitations of the current evidence base should be acknowledged, as they affect the interpretation of both the clinical efficacy and mechanistic findings reviewed here.

The majority of the included clinical studies were single-center, small-sample trials, and most did not employ adequate blinding of participants, practitioners, or outcome assessors, which introduces a potential risk of performance and detection bias. Moreover, many trials combined acupuncture with pharmacotherapy (e.g., corticosteroids, vasodilators, neurotrophic agents), making it difficult to isolate the specific contribution of acupuncture. The possible influence of a placebo effect—arising from the tactile and interpersonal nature of acupuncture—could not be excluded in the absence of well-designed sham-acupuncture controls (70). Finally, spontaneous recovery is a recognized feature of SSNHL (71), and the degree to which the reported improvements reflect the natural history of the disease rather than a treatment-specific effect remains uncertain. Furthermore, for a subset of the included studies, the original publications did not report the necessary summary statistics to calculate between-group effect sizes; in these instances, the clinical meaningfulness of the reported improvements could not be independently assessed.

Although the mechanisms reviewed here—auditory pathway plasticity, limbic–cortical emotional regulation, and neurovascular unit restoration—are supported by converging lines of evidence, most of the direct mechanistic data come from animal models or small observational neuroimaging studies. In many cases, the evidence is indirect or extrapolated from related conditions rather than demonstrated in SSNHL patients receiving acupuncture. Causal relationships between the proposed central changes and hearing recovery have not been established, and longitudinal studies that track neuroimaging biomarkers from the acute phase through recovery are lacking.

The acupuncture protocols across studies varied substantially in terms of modality (manual acupuncture, electroacupuncture, warm acupuncture), acupoint selection, treatment frequency, and duration. This heterogeneity limits the comparability of findings and precludes definitive conclusions about the optimal treatment strategy. Equally important, SSNHL itself is not a homogeneous condition (72), and several patient-level factors may influence treatment response to acupuncture. First, the severity of hearing loss at baseline is a well-established prognostic factor (73): patients with mild to moderate SSNHL generally show higher rates of spontaneous and treatment-induced recovery, whereas those with profound hearing loss tend to have poorer outcomes regardless of intervention. Whether acupuncture offers additional benefit across all severity levels or is more effective in specific strata remains unclear. Second, audiometric configuration (e.g., up-sloping, down-sloping, flat, or total deafness) may differentially affect recovery potential, yet most included studies did not stratify results by audiogram type. Third, time from onset to treatment is a critical determinant of prognosis; early intervention has been associated with better outcomes (74), but whether this reflects a disease-specific time window for acupuncture neuromodulation or simply the natural history of SSNHL has not been disentangled. Fourth, the presence or absence of vertigo is a key clinical distinction (75), as SSNHL with vertigo may indicate more extensive inner ear damage involving both cochlear and vestibular compartments, potentially altering the therapeutic response. Fifth, age and vascular or metabolic comorbidities (e.g., hypertension, diabetes mellitus, hyperlipidemia) may modify both the underlying pathophysiology and the response to acupuncture through their effects on microvascular function and neuroplasticity. Finally, concurrent use of corticosteroids and other pharmacotherapies varied across studies, making it difficult to isolate the effect of acupuncture from that of concomitant treatments.

In summary, current evidence is insufficient to draw firm conclusions about whether acupuncture effects differ meaningfully across these subgroups. Future studies should consider pre-specified subgroup analyses or stratified randomization to address this question directly.

Several additional limitations warrant mention. Publication bias favoring positive results is possible, and negative or neutral findings may be underrepresented in the reviewed literature. The present review was restricted to English- and Chinese-language publications, potentially excluding relevant research in other languages. Furthermore, some included studies did not specify the pharmacotherapy used in the control arm in sufficient detail. These factors should be taken into account when evaluating the strength of the overall evidence.

## 7 Conclusion

SSNHL represents a growing public health challenge, particularly given its rising incidence among younger populations and the limited efficacy of current pharmacological treatments. As a complementary therapy, acupuncture has attracted increasing research attention internationally, owing to its broad applicability. Available reports suggest few adverse events; however, more systematic safety reporting is needed before firm conclusions regarding comparative safety can be drawn.

This narrative review synthesized the literature on acupuncture treatment for SSNHL published between 2020 and 2025, focusing on three core central mechanistic pathways: modulation of auditory pathway plasticity, regulation of the limbic–cortical emotional network, and functional integration of neurovascular units. Preliminary evidence suggests that acupuncture may help ameliorate central functional abnormalities in patients with SSNHL via multi-target pathways. These three mechanisms appear interrelated and potentially synergistic: improved neurovascular unit provides a material foundation for the auditory pathway, while regulating the emotional network influences auditory perception via descending pathways. They provide a framework for understanding the central mechanisms by which acupuncture may exert its therapeutic effects.

Based on the above analysis, future research could be conducted in the following directions. First, a multicenter randomized controlled trial design should be adopted, combined with multimodal imaging techniques such as resting-state functional magnetic resonance imaging (fMRI) and diffusion tensor imaging (DTI). This would allow the longitudinal tracking of the effects of acupuncture on the functional connectivity of auditory networks, the default mode network, and emotion-related brain regions in patients with SSNHL. This would clarify the spatiotemporal dynamics of central neural reorganization. Second, clinical studies should be integrated with animal models. By utilizing techniques such as optogenetics and chemogenetics, the molecular pathways through which acupuncture modulates functional activity in specific brain regions. Although preclinical studies suggest neuroprotective mechanisms of acupuncture treatment, these mechanisms require validation through large-scale, well-designed clinical trials and longitudinal follow-up to assess their disease-modifying potential. Third, studies should be conducted to optimize acupuncture treatment protocols for different subtypes of SSNHL and explore personalized acupuncture strategies based on patients’ symptom characteristics and neuroimaging phenotypes. Fourth, the synergistic mechanisms between acupuncture and other therapies, such as medication and hyperbaric oxygen therapy, should be investigated to provide evidence-based support for the development of integrated treatment models combining traditional Chinese and Western medicine.

In summary, although preliminary progress has been made in understanding the central mechanisms of acupuncture for SSNHL, the current evidence remains limited and does not yet support definitive clinical recommendations. Further high-quality clinical trials and in-depth mechanistic studies are needed to clarify the therapeutic value of acupuncture and its specific neural targets. Such work may ultimately inform evidence-based strategies for the clinical management of SSNHL.
