# Supplementary material for: Acupuncture-related therapy for sudden sensorineural hearing loss: clinical efficacy and central mechanistic insights
Source: Front Neurol. 2026 Aug 6;17:1861797. doi: 10.3389/fneur.2026.1861797 (PMC13490167; doi:10.3389/fneur.2026.1861797)
Supplement: Supplementary file 1 [file Table_1.DOCX]

Table 1: Summary of Clinical Studies on Acupuncture‑related Treatment for SSNHL

| Study | Study Design | Randomization Method | Blinding | Sample Size (T/C) | Treatment Group | Control Group | Intervention | Primary Outcome | Follow-up Duration | Adverse Events | Conclusion |
| --- | --- | --- | --- | --- | --- | --- | --- | --- | --- | --- | --- |
| Zhou and Jin(1) | Retrospective study (case-control) | Not reported | Single-blind (outcome assessors) | T:70 C:70 | Electroacupuncture | Conventional acupuncture | Tinggong (SI19), Ermen (TE21), Yifeng (TE17), auditory area 60 minutes once daily, 3 times per week, for a total of 3 months | Average hearing threshold (AHT) | None | Minor local bleeding (n=4 in treatment group, n=6 in control group), Skin bruise (n=3 in treatment group, n=2 in control group), Needle site pain (n=6 in treatment group, n=5 in control group), no serious adverse events | The electroacupuncture group has a between-group difference of -11.15 dB (95%CI: -13.85 to -8.93, *P*<0.01) in the reduction of average hearing threshold compared with the acupuncture control group. |
| Zhi et al.(2) | Retrospective study (cohort) | Not reported | Not reported | T:158, 212, 201 C: 90, 36, 47 | Acupuncture | Corticosteroids alone | Unspecified | Elevated pure-tone threshold (average hearing threshold improvement in dB HL) | None | Not reported | For combined corticosteroid and acupuncture therapy, the elevated pure-tone threshold values in each subtype of sudden deafness show no statistical difference between treatment groups and control groups (all *P*>0.05). |
| Tao et al.(3) | Two-arm parallel-group RCT | Random number table | Single-blind (outcome assessors and data statisticians) | T:39 C:39 | Transcranial repetitive acupuncture, electroacupuncture, conventional acupuncture | Glucocorticoids + batroxobin + Ginkgo biloba extract | Baihui (GV20), Ningshen (Extra), Auditory Area, Mastoid 1 (Extra), Mastoid 2 (Extra), Tinggong (SI19), Tinghui (SI19), Shangguan (GB3), Wangu (GB12), Fengchi (GB20), Gongxue (Extra), Tianyou (TE16), Waiguan (TE5) 30 minutes once daily; treat for 6 consecutive days followed by 1 day of rest; total treatment duration: 3 weeks | PTA | None | None reported | The acupuncture plus medication group had a post-treatment mean hearing threshold of 29.84±11.01 dBHL, while the medication-only group had 35.80±13.13 dBHL (*P*<0.05) |
| Sun et al.(4) | Two-arm parallel-group RCT | Not reported | Not reported | T:32 C:32 | Acupuncture, bloodletting therapy, and psychological intervention | Dexamethasone Sodium Phosphate Injection, Methylcobalamin Capsules, Ginkgo Biloba Leaf Extract Injection | Affected side: Tinghui (SI19), Yifeng (TE17), Tinggong (SI19), Ermen (TE21), Zhongzhu (TE3), Xiaxi (GB43), Taichong (LR3); bilateral Zusanli (ST36), Sanyinjiao (SP6) Acupuncture manipulation once every 15 minutes; Once daily; 7 days per course; 2 courses total | PTA | 1 month after intervention | Not reported | The intervention group had a hearing improvement rate of 87.5% and a tinnitus improvement rate of 81.5%, while the control group had a hearing improvement rate of 75.0% and a tinnitus improvement rate of 43.75% (both *P* < 0.05). |
| Shi et al.(5) | Retrospective study (cohort) | Not reported | Not reported | T:30 C:30 | Acupuncture | Basic medical treatment (vasodilators, thrombolytics, and other drugs to improve microcirculation; glucocorticoids; neurotrophic agents) | Dazhui (GV14), Fengfu (GV16), C3 paravertebral point, C4 paravertebral point, Fengchi (GB20), Gongxue (Extra), Tinggong (SI19), Yifeng (TE17), Waiguan (TE5), Hegu (LI4), Taichong (LR3), Qiuxu (GB40), Qihai (CV6), Xuehai (SP10), Zusanli (ST36) 20 min; 3 times per week, every other day (rest on Sundays) | PTA | 3 months after intervention | No serious adverse events | The early exposure group (acupuncture intervention within 14 days of onset) had a total effective rate of 80.00% and the late exposure group (acupuncture intervention after 14 days of onset) had a total effective rate of 53.33% (*P*<0.05), with lower THI scores in the early exposure group at post-treatment and follow-up time points (*P*<0.05). |
| Sheng et al.(6) | Two-arm parallel-group RCT | Random number table with sealed opaque envelopes | Single-blind (outcome assessors) | T:39 C:39 | Electroacupuncture | Hyperbaric oxygen therapy, Ginkgo biloba extract tablets, Methylcobalamin tablets | Fengchi (GB20), Gongxue (Extra), Yunting area (Extra), Yifeng (TE17), Tinggong (SI19), Tinghui (SI19), Zhongzhu (TE3), Waiguan (TE5) 60 minutes once daily, 6 times per week, for a total of 4 weeks | PTA | None | Not reported | The mean 250-8000 Hz pure tone threshold of the electro-nape-acupuncture combined hyperbaric oxygen group is lower than that of the hyperbaric oxygen group at 4 weeks post intervention (*P*<0.0001). |
| Qin et al.(7) | Retrospective study (case-control) | Not reported | Not reported | T:154 C:143 | Embedding needle | Intravenous infusion of Ginkgo biloba extract + fibrinolytic enzyme, combined with intravenous infusion of dexamethasone and tympanic cavity injection | Tinghui (GB2), Yifeng (TE17), Ermen (TE21) 1-2 minutes; Needle replacement every 2 days; total treatment duration: 10 days | PTA | None | Not reported | The press needle group had a mean hearing improvement of 19.55±1.72 dB and a response rate of 59.09%, while the control group had a mean hearing improvement of 13.81±1.42 dB and a response rate of 47.55% (*P*=0.005, *P*=0.046). |
| Ma et al.(8) | Prospective cohort study | Not reported | Not reported | T:160 C:160 | Ear Acupuncture | Routine Care | Shenmen (TF_4_), Heart (CO_15_), Kidney (CO_10_), Occipital (AT_3_), Sympathetic (AH_6a_), Inner Ear (LO_6_), Outer Ear (TG_1u_), Endocrine (CO_18_) 3-5 minutes; At least 6 times daily for 15 consecutive days | PTA | None | Redness (7.27%), itching (11.52%), rash (1.82%); Yes | The auricular point sticking group had a cure rate of 30.6% and the non-auricular point sticking group had a cure rate of 18.1%, with between-group differences in hearing recovery (*P*=0.020) and cure rate (*P*=0.009). |
| Lee et al.(9) | Retrospective study (cohort) | Not reported | Not reported | T:37 C:43,74 | Acupuncture | Steroid therapy, Steroids + hyperbaric oxygen therapy | Yifeng (TE17), Tinghui (GB2), Shuigu (GB8), Fengchi (GB20), Xiaguan (ST7), Tinggong (SI19), Zhongzhu (TE3), Houxi (SI3) 10-15 minutes twice weekly for at least 1 month; frequency may be adjusted to 1-3 times weekly based on the degree of hearing recovery | PTA | 2 months after intervention | None reported | The S-H-A group had a 4-frequency pure tone average hearing gain of 38.6±3.9 dB, the S-H group had 25.0±2.6 dB, and the S group had 20.0±3.7 dB (*P*=0.002). |
| Jiang et al.(10) | Four-arm parallel-group RCT | Random number table with sealed opaque envelopes | Single-blind (outcome assessors and data statisticians) | T:30,30,31 C:27 | Electroacupuncture | Conventional drug therapy, Danhong Injection, Vinorelbine Injection, Methylcobalamin Injection | Ermen (TE21), Tinggong (SI19), Tinghui (GB2), Fengchi (GB20), Yifeng (TE17), Zhongzhu (TE3), and Xiaxi (GB43) 30 min once daily, 10 days per course, for a total of 2 courses | PTA | None | Not reported | The 2 Hz/50 Hz dense-dispersion electroacupuncture combined with medication group showed a pure tone hearing threshold total effective rate of 93.5% and a recovery rate of 41.9%, while the medication alone group showed a pure tone hearing threshold total effective rate of 50.0% and a recovery rate of 11.5% (*P*<0.05). |
| Tang and Zhang(11) | Two-arm parallel-group RCT | Not reported | Not reported | T:40 C:40 | Holographic Copper Acupuncture and Scraping Therapy for the Ear | Ginkgo biloba extract, gastrodin injection, dexamethasone, methylcobalamin tablets | Specific auricular points were selected based on the patient's syndrome differentiation results (specific point names were not explicitly listed)  30 minutes; One course of treatment lasts 2 weeks; a total of 2 courses | PTA | None | Not reported | The treatment group had a total effective rate of 97.5%, while the control group had a total effective rate of 77.5% (*P*<0.05). |
| Le et al.(12) | Two-arm parallel-group RCT | Not specified | Not reported | T:84 C:72 | Acupuncture | Batroxobin | Ermen (TE21), Tinggong (SI19), Tinghui (GB2), Yifeng (TF17), Zhongzhu (TE3), Taichong (LR3), Xiaxi (GB43) 30 minutes once daily for 7 consecutive days per course; 2 courses in total, with a 1-day interval between courses | PTA | None | Not reported | The observation group (batroxobin combined with acupuncture) had a total effective rate of 66.67% and a total frequency PTA threshold improvement of 13.9±12.05 dB, while the control group (batroxobin alone) had a total effective rate of 44.44% and a total frequency PTA threshold improvement of 9.22±8.63 dB. |
| Zhang et al.(13) | Two-arm parallel-group RCT | Random number table | Not reported | T:50 C:50 | Acupuncture, electroacupuncture | Conventional drug therapy, methylprednisolone, alprostadil, sodium monosialotetrahexosylganglioside, ginado, lidocaine | Tinggong (SI19), Tinghui (SI19), Yifeng (TE17), Ermen (TE21), Waiguan (TE5), Zhongzhu (TE3), Yanglingquan (GB34), Zulinqi (GB41), Sanyinjiao (SP6); for external invasion by wind-pathogen, add Hegu (LI4), Quchi (LI11), Fengchi (GB20); for liver yang hyperactivity, add Taichong (LR3), Taixi (KI3); for liver fire rising, add Xingjian (LR2), Xiaxi (GB43); for deficiency of qi and blood, add Qihai (CV6), Xuehai (SP10), Zusanli (ST36); for qi stagnation and blood stasis, add Geshu (BL17), Xuehai (SP10) 30 min once daily, 5 times per week, with 2 rest days per week; 10 sessions constitute one course of treatment; a total of 2 courses | PTA | 4 weeks after intervention | Not reported | The observation group had lower post-intervention mean hearing thresholds at 500, 1000, 2000, 4000 Hz than the control group (*F*=6.905, 4.237, 2.063, *P*<0.05). |
| Zhou et al.(14) | Two-arm parallel-group RCT | Not specified | Not reported | T:47 C:47 | Auricular acupuncture, acupoint application | Conventional Western medication, Mecobalamin tablets, Flunarizine hydrochloride tablets | Inner ear (LO_6_), Outer ear (TG_1u_), Kidney (CO_10_), Adrenal gland (TG_2P_), Gallbladder (CO_9_), Yongquan (KI1), Shenque (CV8) 30 minutes once daily | PTA | None | Gastrointestinal discomfort (n=2), skin rash (n=1), pruritus (n=2) | The combined group had a total effective rate of 95.74%, while the conventional group had a total effective rate of 82.98 (*P*<0.05) |
| Li et al.(15) | Two-arm parallel-group RCT | Random number table | Not reported | T:46 C:46 | Acupuncture | Dexamethasone sodium phosphate injection, Ginkgo biloba leaf extract injection | Affected side: Shuaigu (GB8), Qubin (GB7), Ermen (TE21), Tinghui (SI19), Yifeng (TE17); bilaterally: Xuehai (SP10), Taichong (LR3), Zuqiaoyin (GB44) 30 minutes once daily for 10 consecutive days | PTA | None | Local pinhole swelling (n=1), skin itching (n=1), fever (n=1) | The treatment group had a clinical response rate of 97.83% (45/46), while the control group had a rate of 82.61% (38/46; *P*<0.05) for patients with qi-stagnation blood-stasis type sudden deafness. |
| Deng et al.(16) | Two-arm parallel-group RCT | Not specified | Not specified | T:43 C:43 | Acupuncture, intratympanic steroid injection | Intratympanic steroid injection | Tinghui (SI19), Ermen (TE21), Yifeng (TE17), Tinggong (SI19), Zhongzhu (TE3), Taichong (LR3), Hegu (LI4), Taixi (KI3), Waiguan (TE5), Fengchi (GB20), Zusanli (ST36), Xuehai (SP10) 30 minutes per session; Once every other day | PTA | None | Insomnia, facial edema, blood glucose elevation | The combination group (acupuncture plus intratympanic hormone injection) showed post-treatment pure tone hearing thresholds of 35.14±8.35 dB at 500Hz, 37.38±7.42 dB at 1000Hz and 39.72±7.46 dB at 2000Hz, and a clinical effective rate of 97.67%, while the single group (intratympanic hormone injection alone) showed corresponding thresholds of 51.29±9.18 dB, 55.29±5.17 dB, 53.42±5.39 dB and an effective rate of 81.40% (*P*<0.05). |
| Fan et al.(17) | Two-arm parallel-group RCT | Random number table | Not specified | T:68 C:68 | Acupoint injection | Intravenous dexamethasone infusion, betahistine, methylcobalamin tablets, hyperbaric oxygen therapy | Yifeng (TE17) Once daily, for a total of 5 injections over 5 days | PTA | None | Not reported | The treatment group had a hearing efficacy total response rate of 88.24%, while the control group had a rate of 86.76% (*P*>0.05). |
| Huang(18) | Two-arm parallel-group RCT | Not reported | Not specified | T:25 C:25 | Acupoint Injection | Conventional baseline treatment, intravenous injection of methylcobalamin | Ermen (TE21), Tinghui (SI19), Tinggong (SI19), Yifeng (TE17) Once daily, treatment course: 15 days | PTA | 24-48 hours after intervention | Not reported | The treatment group had an overall clinical treatment effectiveness of 92.0% (23/25), while the control group had 64.0% (16/25; P<0.05, χ²=2.713). |
| Guo et al.(19) | Two-arm parallel-group RCT | Random number table | Not reported | T:30 C:30 | Acupuncture | Conventional western medicine treatment, Ginkgo biloba leaf extract injection, methylprednisolone sodium succinate injection | Primary acupoints: Wangu (GB12), Tinggong (SI19), Tinghui (SI19), Yifeng (TE17) on the affected side;  auxiliary acupoints: Xuehai (SP10), Taichong (LR3) on both sides 30 min once daily for 7 consecutive days | Traditional Chinese Medicine Syndrome Scores | None | None reported | The treatment group showed a clinical total effective rate of 86.67% (26/30), while the control group showed a rate of 63.33% (19/30; *P*<0.05). |
| Fan et al.(20) | Two-arm parallel-group RCT | Random number table | Not reported | T:34 C:34 | Zhu Yu Tong Qiao Cong Er Decoction, Acupoint Injection | Standard Western medication, acupoint injection | Ermen (TE21), Tinggong (SI19), Yifeng (TE17), Sanyangluo, Zhigou, Yemeng, Zusanli (ST36), Sanyinjiao (SP6), Zhiyin (BL67), Wangu (GB12) 1 session every 5 days | PTA | None | Not reported | The observation group had a 91.18% total response rate, a 35.45±4.18 dB 0.25 kHz hearing threshold, 4.00±0.38 mPa·s whole blood viscosity, and shorter symptom resolution times than the control group (70.59% total response rate, 42.20±5.05 dB 0.25 kHz hearing threshold, 5.12±0.49 mPa·s whole blood viscosity; all *P*<0.05). |
| Liu et al.(21) | Two-arm parallel-group RCT | Not specified | Not reported | T:40 C:40 | Acupuncture, Hyperbaric Oxygen Therapy | Integrated Traditional Chinese and Western Medicinal Therapy (to improve circulation: Ginkgo biloba extract; to reduce fibrinogen: bacillus coagulans; to nourish nerves: methylcobalamin; Traditional Chinese Medicine modified according to syndrome; glucocorticoids: dexamethasone) | Excess Pattern: Tinghui (SI19), Yifeng (TE17), Zhongzhu (TE3), Xiaxi (GB43), Xingjian (LR2), Qiuxu (GB40); Deficiency Pattern: Taixi (KI3), Shenshu (BL23), Tinggong (SI19), Yifeng (TE17) Once daily | PTA | None | Not reported | The improvement of mean hearing threshold in the treatment group (23.90±12.56 dB) differs from that in the control group (15.90±13.58 dB; *P*=0.010), and the total effective rate in the treatment group (77.5%) differs from that in the control group (55.0%; *P*=0.030) for patients with idiopathic sudden sensorineural hearing loss. |
| Liu et al.(22) | Two-arm parallel-group RCT | Random number table | Not reported | T:39 C:39 | Electroacupuncture, bloodletting | Oral conventional medications (prednisolone acetate tablets, Ginkgo biloba extract tablets, and methylcobalamin capsules) combined with acupuncture and electroacupuncture treatment | Ermen (TE21), Tinggong (SI19), Tinghui (SI19), Yifeng (TE17), Zhongzhu (TE3), Xiaxi (GB43), Fengchi (GB20), Waiguan (TE5), Hegu (LI4), Xingjian (LR2), Qiuxu (GB40), Zulinqi (GB41), Fenglong (ST40), Neiting (ST44) 30 min once daily for 5 consecutive days constitutes one course of treatment; after a 2-day rest period, the next course begins; a total of 4 courses | PTA | None | None reported | The electroacupuncture plus bloodletting group had a pure tone hearing threshold of 42.078±12.074 dB, and the electroacupuncture group had a pure tone hearing threshold of 48.945±12.849 dB after treatment (*P*<0.05). |
| Liu et al.(23) | Two-arm parallel-group RCT | Not specified | Not reported | T:40 C:40 | Acupuncture; vestibular rehabilitation training | Standardized drug therapy, vestibular rehabilitation training | Sphenopalatine ganglion No retention of needles; Once every 3 days, for a total of 5 sessions; 14 days constitute one treatment course | PTA | None | Not reported | The experimental group had a total effective rate of 87.5%, while the control group had a total effective rate of 65.0% (*P*<0.05). |
| Luo et al.(24) | Two-arm parallel-group RCT | Random number table | Not reported | T:60 C:60 | Electroacupuncture, acupoint injection | Acupoint injection of methylprednisolone sodium succinate | Chimai (TE18), Tinghui (SI19), Tinggong (SI19), and Ermen (TE21) 30 minutes once daily | PTA | None | Nausea and vomiting (n=4 in control group, n=4 in observation group), rash (n=1 in control group, n=2 in observation group), dizziness (n=2 in control group, n=2 in observation group), fainting during acupuncture (n=1 in observation group) | The observation group had a total effective rate of 95.0% for hearing and 93.3% for tinnitus, while the control group had 83.3% for hearing and 80.0% for tinnitus (all *P*<0.05). |
| Ma et al.(25) | Two-arm parallel-group RCT | Not specified | Not reported | T:50 C:50 | Acupuncture, Tongqiao Huoxue Decoction | Conventional drug therapy, Mecobalamin tablets, Alprostadil injection | Tinggong (SI19), Tinghui (SI19), Fengchi (GB20), Yifeng (TE17), Zhongzhu (TE3), Yunting area (Extra) 30 minutes once daily | PTA | None | Dizziness (n=1), rash (n=1) | After 4 weeks of intervention, the treatment group had BV-HS of 4.10±0.98 mPa·s, PV of 1.20±0.31 mPa·s, HCT of 37.10±4.52%, total effective rate of 98.00%, and adverse reaction rate of 4.00%, while the control group had corresponding values of 5.10±1.02 mPa·s, 1.52±0.54 mPa·s, 44.02±3.98%, 76.00%, and 36.00% (all *P*<0.05). |
| Nie and Shi(26) | Two-arm parallel-group RCT | Not specified | Double-blind | T:41 C:41 | Acupuncture | Conventional treatment, anticoagulants, glucocorticoids, drugs to improve inner ear microcirculation, blood viscosity-modulating drugs | Primary Acupoints: Unilateral Shenting (GV24), Tinggong (SI19), Yifeng (TE17), and Baihui (GV20); Auxiliary Acupoints: For excess patterns: bilateral Waiguan (TE5), Xingjian (LR2), Qiuxu (GB40), and Zulinqi (GB41); for deficiency patterns: bilateral Neiguan (PC6), Taichong (LR3), Taixi (KI3), and Zhubin (KI9) 30 minutes once daily; 12 days per course; 2 consecutive courses | PTA | None | Not reported | The acupuncture group had a total effective rate of 95.12% and a post-treatment hearing threshold of 27.12±8.12 dB, while the control group had a total effective rate of 78.05% and a post-treatment hearing threshold of 40.17±10.66 dB (*P*<0.05 for both indicators). |
| Sun et al.(27) | Two-arm parallel-group RCT | Random number table | Not reported | T:30 C:30 | Tiao Shen acupuncture, electroacupuncture | Basic treatment (methylcobalamin tablets, Ginkgo biloba extract tablets, and hyperbaric oxygen therapy), combined with electroacupuncture | Control group: bilateral Fengchi (GB20), Gongxue (Extra), affected-side Tinggong (SI19), Tinghui (SI19), and Wangu (GB12); Treatment group: Baihui (GV20), bilateral Benshen (GB13), Neiguan (PC6), Shenmen (HT7), and Sanyinjiao (SP6) 30 min once daily, 5 times per week | PTA | None | No serious adverse events | At 4 weeks post-treatment, the average hearing threshold was 35.73 ± 10.54 dBHL in the treatment group and 44.20 ± 9.76 dBHL in the control group (*P*<0.05). |
| Wang(28) | Two-arm parallel-group RCT | Not specified | Not reported | T:26 C:26 | Acupuncture, nasal air insufflation | Acupuncture alone | Affected side: Ermen (TE21), Tinggong (SI19), Tinghui (SI19), and Yifeng (TE17); both sides: Zhongzhu (TE3), Zusanli (ST36); affected side: Yuji  30 min: 3-week treatment course | PTA | None | Not reported | The observation group had a pure tone hearing threshold of 27.00±5.40 dB, a tinnitus TEQ score of 6.88±2.64, a clinical total response rate of 92.31%, and an ear fullness incidence of 3.85%, while the control group had a pure tone hearing threshold of 31.96±7.09 dB, a tinnitus TEQ score of 9.50±2.20, a clinical total response rate of 84.62%, and an ear fullness incidence of 38.46% (all *P*<0.05). |
| Wang(29) | Two-arm parallel-group RCT | Random number table | Not reported | T:75 C:75 | Warming and penetrating acupuncture technique | Glucocorticoids, neurotrophic agents, hyperbaric oxygen therapy | Fengchi (GB20)  Treatment course: 6 weeks | PTA | None | Not reported | The observation group had a clinical total effective rate of 96.00%, while that of the control group was 84.00% (*P*<0.05). |
| Wang and Huang(30) | Two-arm parallel-group RCT | Random number table | Not reported | T:40 C:40 | Acupuncture using the ‘eight methods of intelligent turtle’ | Conventional western medication treatment: methylprednisolone sodium succinate, bacillus subtilis injection, ginkgo biloba leaf extract injection | Gongsun, Neiguan (PC6), Houxi, Shenmai, Zulinqi (GB41), Waiguan (TE5), Lieque, Zhaohai, Tinghui (SI19), Yifeng (TE17), Xiaxi (GB43), Zhongzhu (TE3) 30 min once every 2 days | PTA | None | None reported | The treatment group had a post-treatment average hearing threshold of (37.00±7.13 dBHL) and a total effective rate of 85.00%, while the control group had a post-treatment average hearing threshold of (41.35±6.81 dBHL) and a total effective rate of 70.00% (*P*<0.05) |
| Xu et al.(31) | Two-arm parallel-group RCT | Random number table | Not reported | T:37 C:37 | Warm Acupuncture | Prednisolone Acetate Tablets Combined with Ginkgo Biloba Leaf Extract Injection | Tinggong (SI19), Tinghui (SI19), Yifeng (TE17), Ermen (TE21) (alternate), Waiguan (TE5), Hegu (LI4), Zusanli (ST36), Sanyinjiao (SP6), Taichong (LR3) 20 min once daily; 5 days constitute one course of treatment; a total of 3 courses | Traditional Chinese Medicine Syndrome Scores | None | Not reported | The observation group showed lower post-treatment traditional Chinese medicine syndrome scores, pure tone audiometry thresholds, whole blood viscosity and hematocrit levels, higher activated partial thromboplastin time, thrombin time and prothrombin time, as well as a 97.30% total effective rate and 59.46% cure rate, compared with the control group which had a 78.38% total effective rate and 29.73% cure rate (all *P*<0.05). |
| Zhang and Chen(32) | Three-arm parallel-group RCT | Not specified | Not reported | T: 60 C: 30 | Hyperbaric oxygen, acupuncture | Conventional drug therapy (vasodilators, agents to improve inner ear microcirculation, neurotrophic agents, glucocorticoids) | Excess pattern: Tinghui (SI19), Yifeng (TE17), Zhongzhu (TE3), Xiaxi (GB43), Xingjian (LR2), Qiuxu (GB40); Deficiency pattern: Taixi (KI3), Shenshu (BL23), Tinggong (SI19), Yifeng (TE17) Once daily for a total of 14 sessions | PTA from baseline | None | Not reported | The acupuncture combined with hyperbaric oxygen group had a post-treatment mean hearing threshold of 34.03±13.60 dB, total effective rate of 83.33%, while the control group had 50.27±14.23 dB and 46.67% respectively (*P*<0.05) |
| Zhang et al.(33) | Two-arm parallel-group RCT | Random number table | Not specified | T:48 C:48 | Auricular acupressure, hyperbaric oxygen | Conventional treatment (microcirculation optimization, edema reduction, nerve nutrition) + Auricular acupressure, Vitamin B1 injection, Methylcobalamin injection, Ginkgo biloba leaf extract injection | Kidney point, Inner Ear point, Outer Ear point 1-2 minutes; 2-3 times daily | PTA | 30 days after intervention | Burning sensation (n=2), ear pain (n=1), fatigue (n=1), tinnitus (n=2), muscle soreness (n=2) | The treatment group had a total effective rate of 95.83%, while the control group had a total effective rate of 83.33% (*P*<0.05) |
| Zhang et al.(34) | Retrospective study (cohort) | Not reported | Not specified | T:34 C:48 | Acupuncture | Bacillus subtilis enzyme injection, conventional acupuncture, neurotrophic therapy, and microcirculation improvement therapy | Acupoints around the ear: Yifeng (TE17), Ermen (TE21), Tinggong (SI19), Acupoint selection based on meridian diagnosis 30 min once daily | PTA | 14 days after intervention | Not reported | The RSHL group had a total effective rate of 30.23%, while the control group had a total effective rate of 39.68% (*P*<0.05). |
| Zhang et al.(35) | Two-arm parallel-group RCT | Random number table | Not reported | T:33 C:33 | Acupuncture at Four Periaural Points | Conventional pharmacotherapy (IV methylprednisolone, betahistine, lidocaine; PO mecobalamin, flunarizine, Ginkgo biloba) plus TCM syndrome-based herbal formula | Wangu (GB12), Tinggong (SI19), Tinghui (GB2), Yifeng (TE17) 30 minutes; 7-day treatment course | PTA | Immediately after intervention | None reported | The observation group had a hearing total effective rate of 96.97% and a tinnitus total effective rate of 100.00%, while the control group had a hearing total effective rate of 81.82% and a tinnitus total effective rate of 87.88% (*P*<0.05). |
| Zhang et al.(36) | Two-arm parallel-group RCT | Random number table | Not reported | T:60 C:60 | Transcutaneous acupoint stimulation using a medium-frequency electrotherapy device and intratympanic injection of methylprednisolone | Intratympanic injection of methylprednisolone to improve microcirculation and nourish the nerves | Tinggong (SI19), Yifeng (TE17), and Zhongzhu (TE3) 45 minutes twice daily | PTA | None | Not reported | The observation group had a total effective rate of 95.00% and a 4-week post-treatment mean hearing threshold of (28.65±3.11 dBHL), while the control group had a total effective rate of 83.33% and a 4-week post-treatment mean hearing threshold of (34.27±3.31 dBHL; *P*=0.040, *P*<0.05 respectively). |
| Zhang et al.(37) | Two-arm parallel-group RCT | Random number table | Double-blind (participants, outcome assessors, and statisticians); sham acupuncture control | T:33 C:33 | Acupuncture, electroacupuncture | Sham acupuncture and sham electroacupuncture | Seven-line method of the neck (first line: Du Meridian at Dazhui (GV14); second and third lines: bilateral fourth cervical paravertebral points on the cervical paravertebral lines; fourth and fifth lines: the intersection of the horizontal line at the midpoint of the line connecting Fengchi (GB20) and Gongxue (Extra) on the first lateral line of the Bladder Meridian of the Foot-Taiyang with the prominence of the trapezius muscle at the nape; sixth and seventh lines: bilateral Fengchi (GB20) and Gongxue (Extra) on the Gallbladder Meridian of the Foot-Shaoyang), periauricular acupoints (Tinggong (SI19), Yifeng (TE17)) 30 minutes 3 times per week for 6 consecutive weeks (18 sessions total) | PTA | 4 weeks after intervention | Subcutaneous hematoma (n=2), severe pain(n=5), needle fainting (n=1) | The treatment group showed posttreatment and follow-up impaired frequency pure tone average hearing thresholds (50.57±18.07 dB, 47.70±17.42 dB) and corresponding pre-post change values lower than those in the control group (54.38±21.77 dB, 53.36±20.99 dB; *P*<0.05). |
| Huang et al.(38) | Two-arm parallel-group RCT | Random number table | Not reported | T:37 C:39 | Acupuncture, Modified Cong’er Ximing Decoction | Modified Cong’er Ximing Decoction | Between the Tinggong (SI19) and Tinghui (SI19) acupoints on the affected ear side 20-30 min once a week, for a total of 6 weeks (6 sessions) | PTA from baseline | None | Not reported | The observation group (51.86±19.08 dB) had a post-treatment pure tone threshold different from the control group (60.05±16.35 dB; *P*<0.05). |
| Jiang and Xie(39) | Two-arm parallel-group RCT | Random number table | Not reported | T:32 C:31 | Acupuncture, Tuina, Western Medicine | Dexamethasone Injection, Vincristine Injection, Methylcobalamin Tablets | Ermen (TE21), Tinggong (SI19), Tinghui (SI19), Xiaguan (ST7), Yifeng (TE17), Shuaigu (GB8), Zhongzhu (TE3), Xiaxi (GB43) 30 minutes once daily | PTA | None | Not reported | The observation group had a post-treatment mean hearing threshold of 32.42±17.21 dB, while the control group had 41.22±16.34 dB (*P*<0.05). |
| Liu et al.(40) | Two-arm parallel-group RCT | Random number table | Not specified | T:39 C:39 | Acupuncture | Oral administration of *Tongqiao Huoxue Decoction* (a traditional Chinese herbal formula detailed in the original study) | Affected side: Shuaigu (GB8), Qubin (GB7), Tinghui (SI19), Ermen (TE21), Yifeng (TE17); both sides: Xuehai (SP10), Taichong (LR3), Zuqiaoyin (GB44) Needle retention in the head acupoints for 40 minutes; Needle retention in Tinghui (SI19), Ermen (TE21), and other points for 30 minutes Once daily; 10-day treatment course; 2 treatment courses in total, with a 1-week interval between courses | PTA | None | Not reported | Observation group had a 15-day post-treatment hearing threshold of 37.09±5.71 dB, while control group had 45.26±5.86 dB (*P*<0.05). |
| Wang and Sun(41) | Two-arm parallel-group RCT | Random number table | Not reported | T:60 C:60 | Auricular therapy, Mindfulness Training | Routine nursing combined with mindfulness training | Shenmen (TF_4_), Heart (CO_15_), Liver (CO_12_), Spleen (CO_13_), Kidney (CO_10_) 20 min; pressure application 4-5 times daily; alternate sides every 2 days; treatment duration is 10 days | SAS, SDS, PSQI | None | No serious adverse events | The observation group had SAS score of 41.85±3.64, SDS score of 44.02±5.17, and PSQI total score of 6.51±1.15, while the control group had corresponding scores of 43.60±5.49, 46.17±4.69, and 7.30±2.32 (all *P*<0.05). |
| Wang (42) | Two-arm parallel-group RCT | Random number table | Not reported | T:52 C:50 | Auricular Acupuncture | Alprostadil Dry Emulsion for Injection Combined with Xuesaitong Injection | Affected-side Inner ear (LO_6_), Kidney (CO_10_), Liver (CO_12_), Shenmen (TF_4_), and Subcortical (AT_4_) 10 minutes once daily | Hearing threshold | 1 month after intervention | None reported | The observation group had a total effective rate of 96.1% and a post-treatment hearing threshold of 26.68±4.36 dB, while the control group had a total effective rate of 72.0% and a post-treatment hearing threshold of 38.98±6.69 dB (*P*<0.05). |
| Wang et al.(43) | Two-arm parallel-group RCT | Random number table | Not reported | T:38 C:37 | “Regulating the Spirit and Opening the Orifices” Acupuncture Technique | Conventional Acupuncture Method | Yintang (GV24+), Baihui (GV20), Neiguan (PC6), Tinggong (SI19), Yifeng (TE17), Taichong (LR3), and Taixi (KI3)  30 min once daily; 10 days per course; 2 consecutive courses (with a 2-day break between courses) | Hearing loss score | None | Not reported | The treatment group had a hearing loss score of 1.00 (0, 2.00) and a mean hearing threshold of 29.50 (23.58, 50.08) dB, while the control group had a hearing loss score of 2.00 (1.00, 2.50) and a mean hearing threshold of 43.00 (36.00, 59.83) dB, with a between-group difference of *P*<0.05. |
| Wei and Sun(44) | Two-arm parallel-group RCT | Random number table | Not reported | T:30 C:30 | ‘Regulating and harmonizing the spirit’ Acupuncture Technique, Hyperbaric Oxygen Therapy | Hyperbaric oxygen therapy, corticosteroids, and neurotrophic agents | Baihui (GV20), Shenting (GV24), Yintang (GV24+), Yaoyangguan (GV3), Mingmen (GV4), Fengchi (GB20), Neiguan (PC6), Shenmen (HT7) 30 min; 6 times per week; 4 weeks per course | PTA | None | None reported | The observation group had a total effective rate of 90.0% and a post-treatment pure tone hearing threshold of (41.38±6.94 dB), while the control group had a total effective rate of 76.7% and a post-treatment pure tone hearing threshold of (48.72±7.53 dB; P<0.05 for total effective rate, *P*<0.01 for pure tone hearing threshold) |
| Wu and Cheng(45) | Two-arm parallel-group RCT | Random number table | Not reported | T:44 C:48 | Acupoint application, auricular therapy | Routine Treatment and Care | Yongquan (KI1); Sanjiao (CO_17_), Liver (CO_12_), Kidney (CO_10_), Shenmen (TF_4_), Inner Ear (BL_59_), Subcortical (AT_4_) Acupoint application: 2-3 minutes, once daily at night; auricular therapy: 1-2 minutes, replaced every 3 days | PTA | None | Not reported | The observation group had an 84.09% total effective rate of deafness treatment, while the control group had a 50.00% rate, with *Z*=4.005 and *P*<0.001. |
| Yu et al.(46) | Two-arm parallel-group RCT | Not specified | Not reported | T:31 C:31 | Acupuncture, hyperbaric oxygen | Hyperbaric oxygen combined with nose-pinching and mouth-closing breathing exercises, oral administration of methylcobalamin tablets | Baihui (GV20), Sishencong (EX-HN1), Tinggong (SI19), Tinghui (SI19), Yifeng (TE17), Shuaigu (GB8), Zusanli (ST36), Fenglong (ST40), Taixi (KI3) 20 min once daily; 10 sessions per course; 3 courses in total | PTA | 1 month after intervention | Not reported | The observation group had a hearing total effective rate of 96.77%, while the control group had a hearing total effective rate of 80.65% (*P*<0.05) |
| Zhang et al.(47) | Two-arm parallel-group RCT | Not specified | Not reported | T:52 C:51 | ‘Eight Methods of Intelligent Turtle’ Acupuncture Technique | Conventional Western medication, standard acupuncture | Xiaxi (GB43), Yifeng (TE17), Waiguan (TE5), Zhongzhu (TE3), Zulinqi (GB41), Tinghui (SI19)  30 min once daily for 4 consecutive weeks | PTA | None | Not reported | The observation group had a total effective rate of 96.2%, a post-treatment hearing threshold of 1.13±0.12 dB, and a deafness score of 29.75±3.24, while the control group had a total effective rate of 80.4%, a post-treatment hearing threshold of 1.92±0.31 dB, and a deafness score of 42.11±4.73 (all *P*<0.05). |
| Zhong and Wang(48) | Two-arm parallel-group RCT | Random number table | Not reported | T:40 C:40 | Acupoint injection | Intravenous infusion of dexamethasone sodium phosphate, intravenous infusion of alprostadil, intravenous infusion of methylcobalamin | Chimai (TE18)  Once daily for 2 consecutive weeks | PTA | None | Pruritus (n=2 in control group, n=1 in observation group), fever (n=1 in control group) | The observation group showed mean pure tone hearing thresholds of 46.27±10.59 dB after 1 week of treatment and 37.46±9.73 dB after 2 weeks, while the control group showed 53.03±9.71 dB and 45.26±9.25 dB respectively (*P*<0.05). |
| Duan(49) | Two-arm parallel-group RCT | Random number table | Not reported | T:37 C:35 | *Fucong Decoction* (a traditional Chinese herbal formula detailed in in the original study), acupuncture | Ginardol Injection, Dexamethasone Sodium Phosphate Injection | Zhongzhu (TE3) 30 minutes once daily, for a 14-day course | PTA | None | Not reported | The observation group had a total effective rate of 91.9% (34/37), while the control group had a total effective rate of 74.3% (26/35; *P*<0.05). |
| Fan et al.(50) | Three-arm parallel-group RCT | Random number table | Not reported | T: 35 C: 70 | Acupuncture; Hyperbaric oxygen; Acupuncture combined with hyperbaric oxygen | Basic drug therapy, hyperbaric oxygen, acupuncture | Main Acupoints: Baihui (GV20), affected-side Ermen (TE21), Tinggong (SI19), Tinghui (SI19), Xiaguan (ST7); for excess patterns: add bilateral Zhongzhu (TE3) and Waiguan (TE5); for deficiency patterns: add bilateral Taixi (KI3) and Shenshu (BL23) 30 minutes; 5 times per week for 4 consecutive weeks | PTA | None | Not reported | Group C (acupuncture combined with hyperbaric oxygen) had a mean low-frequency hearing threshold improvement of 27.74±12.53 Db (*P*=0.016). Group A (hyperbaric oxygen alone) had 21.09±10.24 dB and Group B (acupuncture alone) had 20.54±11.3 dB (*P*=0.010). |
| Hu et al.(51) | Two-arm parallel-group RCT | Random number table | Not reported | T:60 C:60 | Acupoint injection | Oral prednisone acetate tablets, ginkgolide drops, and methylcobalamin tablets | Tinggong (SI19), Yifeng (TE17), Neiguan (PC6), Waiguan (TE5) 1 time/day | PTA | None | None reported | The post-intervention mean hearing threshold of the treatment group was lower than that of the control group (*P*<0.05), and the total clinical effective rate was 90.79% in the treatment group and 77.03% in the control group (*P*<0.05). |
| Hu et al.(52) | Two-arm parallel-group RCT | Random number table | Not reported | T:48 C:48 | Acupoint injection | Prednisone tablets combined with Ginkgo biloba extract and methylcobalamin tablets | Tinggong (SI19), Yifeng (TE17), Neiguan (PC6), Waiguan (TE5) Once daily, 5 days per course, for a total of 2 courses | PTA | None | Not reported | The treatment group showed a 91.07% total effective rate and lower hearing thresholds across tested frequencies, while the control group had a 75.44% total effective rate (both *P*<0.05). |
| Huang(53) | Two-arm parallel-group RCT | Random number table | Not reported | T:39 C:39 | Acupuncture | Methylprednisolone sodium succinate, Methylcobalamin tablets | Shuaigu (GB8), Qubin (GB7), Ermen (TE21), Tinghui (GB2), Zuqiaoyin (GB44), Xuehai (SP10), Taichong (LR3) 0.5 hour once daily; 10 days per course; after 2 courses, rest for 5 days; total of 2 courses | PTA | None | No serious adverse events | The acupuncture group had a total effective rate of 97.44% compared with 76.92% in the Western medicine group (*P*<0.05). |
| Jiang(54) | Two-arm parallel-group RCT | Not reported | Not specified | T:15 C:15 | Herbal medicine, acupuncture | Prostaglandin E2 Injection, Methylcobalamin Capsules, Dexamethasone | The same side: Wangu (GB12), Ermen (TE21), Tinggong (SI19), and Tinghui (SI19)  Both sides: Zhongzhu (TE3), Waiguan (TE5), Hegu (LI4), Zusanli (ST36), Sanyinjiao (SP6), and Taichong (LR3) 30 minutes once daily | PTA | None | Not reported | The treatment group had a 93.33% total effective rate of clinical outcomes, while the control group had a 60.00% total effective rate (*P*<0.05). |
| Liao et al.(55) | Two-arm parallel-group RCT | Random number table | Not specified | T:34 C:34 | Acupoint Injection | Intravenous infusion of 10% glucose solution with dexamethasone | Ermen (TE21), Yifeng (TE17), Tinggong (SI19), Tinghui (SI19) Once daily; 1 week constitutes 1 treatment course; rest for 5-7 days between courses; a total of 2 treatment courses | PTA | None | Not reported | The treatment group had a clinical total response rate of 94.12% (32/34), while the control group had a rate of 73.53% (25/34; P<0.05) among sudden deafness patients. |
| Liao and Hua(56) | Two-arm parallel-group RCT | Random number table | Not reported | T:21 C:21 | Acupuncture, Glucocorticoids | Combined glucocorticoid and drug therapy, dexamethasone, bacitracin, Ginaton | Fengchi (GB20), Baihui (GV20) on the head, bilateral Taiyang (EX-HN5), bilateral Tinggong (SI19), bilateral Tinghui (SI19), bilateral vertigo-hearing area 30 minutes per session; 1 session/day | PTA | None | Not reported | The observation group showed a clinical total effective rate of 76.2%, while the control group showed 57.1% (P<0.05) among patients with sudden deafness accompanied by vertigo. |
| Zhou et al.(57) | Two-arm parallel-group RCT | Random number table | Not reported | T:32 C:33 | Needle-embedding therapy | Injection into Periauricular Acupoints | Quchi (LI11), Waiguan (TE5), Ermen (TE21), Tinghui (SI19), Tinggong (SI19), Yifeng (TE17) Once daily for 7-10 consecutive days (1 treatment course) | PSQI | None | Not reported | The observation group (acupoint injection plus press needle therapy) had lower PSQI subscale scores and a lower pure tone average threshold (26.37±3.18 dB) than the control group (acupoint injection alone) at 1 week post-intervention (all *P*<0.05). |
| Song et al.(58) | Three-arm parallel-group RCT | Not specified | Not specified | T:49 C:23 | Acupuncture of Deep needling, conventional acupuncture, and decoction of Chinese herbal medicine | Acupuncture of Deep needling + herbal decoction, Conventional acupuncture + herbal decoction, Oral herbal decoction | Tinghui (SI19), Ermen (TE21), Tinggong (SI19), Yifeng (TE17), Shuaigu (GB8), Chimai (TE18), Fengchi (GB20), Scalp Acupuncture (Area of Auditory Deafness), Zhongzhu (TE3), Waiguan (TE5) | PTA | 1 and 3 months after treatment completion | Not reported | The mean reduction in SAS score was 4.35 in the common acupuncture group, 2.35 in the deep acupuncture group, and 2.17 in the Chinese medicine group; the mean reduction of pure-tone threshold from baseline was 11.31 dB HL in the deep acupuncture group, 10.00 dB HL in the common acupuncture group, and 8.54 dB HL in the Chinese medicine group.. |
| Sun(59) | Two-arm parallel-group RCT | Random number table | Not reported | T:50 C:50 | Acupuncture | Conventional Western medication, including vasodilators, corticosteroids, and neurotrophic agents, for patients with tinnitus, 2% lidocaine hydrochloride was additionally administered via injection | Ermen (TE21), Tinggong (SI19), Tinghui (SI19), Yifeng (TE17), Taichong (LR3), Qiuxu (GB40), Zhongzhu (TE3) 30 minutes once daily; 10 days per course | Hearing loss score | None | Not reported | The clinical total effective rate at 1 and 3 months after treatment was 90.00% (45/50) and 96.00% (48/50) in the study group, and 70.00% (35/50) and 80.00% (40/50) in the control group (*P*<0.05). |
| Wang et al.(60) | Two-arm parallel-group RCT | Random number table | Not reported | T:45 C:45 | Acupuncture | Methylcobalamin Injection Combined with Alprostadil Injection | bilateral Fengchi (GB20), Tianzhu (BL10), Tianyou (TE16)  30 min once daily, 5 times per week | PTA | None | Not reported | The observation group had a post-treatment hearing loss score of 1.22±0.17, hearing threshold of 29.40±4.44 dB, and clinical total effective rate of 93.33%, while the control group had 1.73±0.25, 43.77±5.91 dB, and 75.56% respectively (*P*<0.05 or *P*<0.01). |
| Ye et al.(61) | Two-arm parallel-group RCT | Random number table | Not reported | T:117 C:136 | Acupuncture, hyperbaric oxygen | Conventional drug therapy, acupuncture | Primary Acupoints: Baihui (GV20), Yifeng (TE17), Shenting (GV24), Tinghui (SI19), Tinggong (SI19); Auxiliary Acupoints: For excess patterns: add Waiguan (TE5), Xingjian (LR2), Qiuxu (GB40); for deficiency patterns: add Neiguan (PC6), Taixi (KI3), Taichong (LR3) 30 minutes once daily; 7 days per course | Hearing threshold | 1 year | Ear distending pain (n=24), external auditory canal redness and swelling (n=1), insomnia (n=5) | Group B (hyperbaric oxygen combined with acupuncture) had a post-treatment mean hearing threshold of 24.72±17.74 Hz and a total effective rate of 79.49%, while Group A (acupuncture alone) had a post-treatment mean hearing threshold of 44.38±27.29 Hz and a total effective rate of 40.44% (*P*<0.01 for hearing threshold, *P*<0.05 for total effective rate). |
| Yu and Sun(62) | Two-arm parallel-group RCT | Random number table | Not reported | T:24 C:24 | Periauricular acupoint injection, periauricular massage | Intratympanic injection of methylprednisolone sodium succinate, periauricular massage | Tinggong (SI19), Tinghui (SI19), Ermen (TE21), Yifeng (TE17) Once every other day, for a total of 5 injections; 10 days constitute one treatment course | Hearing threshold | None | Not reported | The observation group had a post-treatment hearing threshold of (19±7) dB, while the control group had a post-treatment hearing threshold of (27±10) dB (*P*<0.05). |
| Zhang et al.(63) | Two-arm parallel-group RCT | Random number table | Not reported | T:40  C:40 | Fang’s scalp acupuncture, acupoint injection | Methylcobalamin acupoint injection | Head (2 cm anterior to the coronal-sagittal point, 2 cm wide on both sides), Upper Jiao of the Inverted Zang Organs (4 cm below the central posterior gyrus), Hearing (1.5 cm above the tip of the ear), Memory (with the sagittal suture as the vertex, draw two straight lines extending 7 cm to the left and right, respectively, at a 60°angle to the sagittal suture), Smell and Taste (3 cm anterior to the tip of the ear); all points are selected bilaterally; The affected side: Yifeng (TE17) and Tinggong (SI19)  30 minutes once daily | Hearing threshold | 3 months after treatment completion | Stomachache (n=2 in control group, n=1 in observation group), nausea (n=3 in control group, n=2 in observation group), loss of appetite (n=1 in control group and observation group) | The treatment group had a total effective rate of 87.50%, a post-treatment traditional Chinese medicine syndrome score of 3.65±1.01, a post-treatment pure tone audiometric threshold of 41.87±7.25 dB and a 0-grade tinnitus proportion of 52.50%, while the control group had corresponding values of 65.00%, 7.24±1.33, 49.13±8.02 dB and 30.00% (*P*<0.05). |
| Zhou et al.(64) | Two-arm parallel-group RCT | Systematic randomization | Not reported | T:79 C:79 | “Opening the Orifices and Restoring Hearing” Acupuncture Technique, Moxibustion | Conventional Western medication treatment: methylprednisolone sodium succinate, alprostadil, Monosialotetrahexosylganglioside sodium, Ginaton, 2% lidocaine, Batroxobin | Baihui (GV20), Yintang (GV24+) (bilateral), Tinggong (SI19) on the affected side, Yifeng (TE17), Shuaigu (GB8), Quanliao (SI18), Waiguan (TE5), Hegu (LI4), Zhongzhu (TE3), Taixi (KI3), Taichong (LR3), bilateral Xuehai (SP10), Zusanli (ST36), Sanyinjiao (SP6) 30 min once every 2 days | Hearing threshold | None | Not reported | The treatment group (acupuncture plus conventional medication) showed an effective rate of 87.34% and a 2-course post-treatment pure tone audiometry threshold of 17.45±2.58 dB, while the control group (conventional medication alone) showed an effective rate of 72.15% and a 2-course post-treatment pure tone audiometry threshold of 26.45±2.36 dB (*P*<0.05). |
| Zhou(65) | Two-arm parallel-group RCT | Random number table | Not reported | T:53 C:52 | Acupuncture | Conventional drug therapy, lifestyle guidance, and psychological counseling | Tinghui (SI19) (affected side), Tinggong (SI19), Ermen (TE21), Zhongzhu (TE3), Yifeng (TE17), Xuehai (SP10), Fengchi (GB20), Waiguan (TE5), Taichong (LR3), Taixi (KI3), Zusanli (ST36), Hegu (LI4) 30 minutes once every other day | PTA | None | No serious adverse events | The experimental group had a treatment total effective rate of 90.57%, while the conventional group had a rate of 75.00% (*P*<0.05). |
| Gao et al.(66) | Three-arm parallel-group RCT | Random number table | Not reported | T:30 C:30 | Acupoint injection (Methylprednisolone) | Methylcobalamin tablets | Acupoint behind the ear Once every other day | PTA | None | None reported | The pure tone hearing thresholds at low frequency, intermediate frequency and high frequency in the treatment group were (31.83±6.94) dB, 34.50 (27.00, 37.00) dB and (24.73±4.78) dB respectively, while those in the control group were (33.77±7.68) dB, 34.00 (27.75, 41.25) dB and (31.96±1.68) dB respectively. |
| Yu et al.(67) | Two-arm parallel-group RCT | Stratified Random Sampling | Single-blind (outcome assessors and data statisticians) | T:110 C:55 | Electroacupuncture, Acupoint Injection | Pharmacological treatment | Ermen (TE21), Tinggong (SI19), Yifeng (TE17) 30 minutes once daily | PTA | None | Not reported | The electroacupuncture group had a total effective rate of 87.3% for sudden deafness after 10 days of treatment, while the acupoint injection group had 67.3% and the control group had 63.6% (*P*=0.014). |
| Yang et al.(68) | Two-arm parallel-group RCT | Random number table | Not reported | T:121 C:107 | Acupuncture, hyperbaric oxygen therapy, and drug therapy | Acupuncture, Methylcobalamin Injection, Ginkgo Biloba Leaf Extract Injection, Dexamethasone Sodium Phosphate Injection | Yifeng (TE17), Ermen (TE21), Tinghui (SI19), Taichong (LR3), Zhongzhu (TE3) 30 minutes once daily; 7 sessions constitute one course of treatment | SAS, SDS | None | None reported | The treatment group had a response rate of 68.6% and an overall effective rate of 85.1%, while the control group had a response rate of 57.9% and an overall effective rate of 71.0% (*P*<0.05). |
| Huang et al.(69) | Two-arm parallel-group RCT | Random number table | Not reported | T:28 C:28 | Auricular therapy and community health education intervention | Auricular point pressing | Liver (CO_12_), Kidney (CO_10_), Inner Ear (LO_6_), Endocrine (CO_18_), Subcortical (AT_4_), Shenmen (TF_4_) 1-2 minutes Pressing 5 times daily; replacing once every 4 days | SAS, SDS | None | Not reported | The combined group had SAS score of (24.45±1.03), SDS score of (24.31±1.41), and total nursing satisfaction of 96.43%, while the control group had SAS score of (35.36±1.14), SDS score of (36.64±1.48), and total nursing satisfaction of 64.29% (all *P*<0.05). |
| Zhang et al.(70) | Three-arm parallel-group RCT | Random number table | Not reported | T:42 C:42 | Auricular point pressing | Progressive muscle relaxation training | Subcortical (AT_4_), Shenmen (TF_4_), Kidney (CO_10_), External Ear (TG_1u_), Inner Ear (LO_6_), Spleen (CO_13_), Stomach (CO_4_), Liver (CO_12_) 1-2 minutes;3-5 times daily | SAS | None | No serious adverse events | The experimental group had SAS score (50.01±4.92), SDS score (55.02±5.21), air conduction hearing threshold (27.61±9.74 dBHL), hearing loss decibel (26.68±6.72 dB) and total effective rate (95.24%), while the control group had SAS score (54.33±5.58), SDS score (58.01±5.24), air conduction hearing threshold (36.22±11.71 dBHL), hearing loss decibel (32.14±9.43 dB) and total effective rate (80.95%) (all *P*<0.05). |

**References**

1.	Zhou X-F, Jin X-L. Effectiveness of electroacupuncture for the treatment of sudden sensorineural hearing loss: A retrospective study. *Medicine (Baltimore)* (2021) 100:e25665. doi: 10.1097/MD.0000000000025665

2.	Zhi L, Li S, Feng Y, Liu Q, Jiang Z, Zhong C, He Y. Effect of corticosteroid-based combination therapy on sudden deafness: a retrospective study. *Am J Otolaryngol* (2025) 46:104666. doi: 10.1016/j.amjoto.2025.104666

3.	TAO R, ZUO X, BAI S, ZHU P. Repeated transcranial acupuncture combined with electroacupuncture for flat descending sudden deafness: a randomized controlled trial. *Chinese Acupuncture & Moxibustion* (2023) 43:932–936. doi: 10.13703/j.0255-2930.20230223-k0002

4.	Sun H, Chu C, Wang X, Gao F. The psychological intervention and clinical efficacy of Rosenthal effect and acupuncture bleeding on middle-aged and young patients with sudden hearing loss. *Medicine (Baltimore)* (2024) 103:e36820. doi: 10.1097/MD.0000000000036820

5.	SHI A, LI Y, ZHANG J, XING Y, HONG Y, BAI P. Effect of acupuncture intervention time on the therapeutic effect in patients with sudden hearing loss. *Acupuncture Research* (2024) 49:618–624. doi: 10.13702/j.1000-0607.20230299

6.	Sheng G-B, Su H, Li H-L, Bao R, Liu G, Kong Y, Tang Y. [Effect of electro-nape-acupuncture on hearing in patients with refractory flat descending idiopathic sudden sensorineural hearing loss]. *Zhongguo Zhen Jiu* (2020) 40:726–730. doi: 10.13703/j.0255-2930.20190614-k0002

7.	Qin F, Guo S, Yin X, Guo J, Lu X, Ma J. Treatment of idiopathic sudden sensorineural hearing loss with press needle. *J Pak Med Assoc* (2025) 75:1199–1203. doi: 10.47391/JPMA.20232

8.	Ma D, Qian Q, Fan Y, Zuo Y, Ye L, Chen Y. Auricular Point Sticking for Patients with Sudden Sensorineural Hearing Loss: A Propensity Score Matching Analysis. *Int J Gen Med* (2025) 18:4079–4089. doi: 10.2147/IJGM.S527833

9.	Lee C-J, Chen H-C, Shih C-P, Lin Y-Y, Kuo C-Y, Liu S-C. Combined Acupuncture-Hyperbaric Oxygen-Steroids Therapy for Idiopathic Sudden Sensorineural Hearing Loss: A Retrospective Observational Study. *J Altern Complement Med* (2021) 27:588–595. doi: 10.1089/acm.2020.0330

10.	Jiang S-Y, Hou W-Z, Ni G-X, Jiang Z-Q. [Effect of different electroacupuncture stimulation parameters on sudden hearing loss]. *Zhongguo Zhen Jiu* (2021) 41:1103–1107. doi: 10.13703/j.0255-2930.20200913-k0001

11.	Tang Z, Zhang L. Effect of mecobalamin combined with holographic copper acupuncture and scraping technique on tinnitus symptoms in patients with sudden sensorineural hearing loss. *Trop J Pharm Res* (2024) 23:761–768. doi: 10.4314/tjpr.v23i4.12

12.	Le X, Liu J, Jia Q, Wu J, Jiang Y, Lv Z. Effect of combining batroxobin with acupuncture treatment on hearing recovery in patients with sudden sensorineural hearing loss. *Acta Oto-Laryngol* (2024) 144:299–305. doi: 10.1080/00016489.2024.2369295

13.	ZHANG W, CAI W, LIANG J, CHEN P. The Effect of Acupuncture on Quality of Life and Anxiety and Depression in Patients with Sudden Sensorineural Hearing Loss. *Chinese Journal of Otology* (2020) 18:1060–1065.

14.	ZHOU Q, LI J, FENG F, MIN L, ZHONG W. Curative effect of auricular acupuncture combined with acupoint application on sudden deafness and its influences on hemorheology, hearing level and nail -fold microcirculation. *Journal of Sichuan of Traditional Chinese Medicine* (2023) 41:220–223.

15.	Li Q, Cao Y, Hu Z. A Clinical Study on the Treatment of 46 Cases of Sudden Deafness of the Qi Stagnation and Blood Stasis Type with Acupuncture Combined with Conventional Therapy. *Jiangsu Journal of Traditional Chinese Medicine* (2023) 55:48–52. doi: 10.19844/j.cnki.1672-397X.2023.09.012

16.	Deng Q, Chen C, Yu L. Acupuncture combined with tympanic cavity injection of hormones in the treatment of sudden deafness. *Chinese Medical Digest Otorhinolaryngology* (2023) 38:107–109. doi: 10.19617/j.issn1001-1307.2023.01.107

17.	FAN Y, HUANG W, ZHANG R, HAO Y. Efficacy and safety of dexamethasone acupoint injection in treatment of sudden deafness. *Journal of Tongji University(Medical Science)* (2024) 45:106–111.

18.	Huang H. The effect of methocobalamin injection on intravenous injection on sudden deafness. *Chinese Medical Digest Otorhinolaryngology* (2023) 38:39–41. doi: 10.19617/j.issn1001-1307.2023.01.39

19.	GOU C, LI Y, ZHANG Y, WEI D. Clinical Efficacy of Acupuncture in Treating Qi Stagnation and Blood Stasis Type Sudden Deafness Based on the Theory of “Xuanfu-Mailuo.” *Information on Traditional Chinese Medicine* (2025) 42:52–56. doi: 10.19656/j.cnki.1002-2406.20250609

20.	FAN Q, QI G, WANG L, LI S. Clinical Observation on Zhuyu Tongqiao Cong’er Decoction Combined with Acupoint Injection in the Treatment of Deafness of Qi Stagnation and Blood Stasis Type. *Medical Innovation of China* (2022) 19:133–136.

21.	LIU D, LIU Y, ZHANG X. Efficacy Analysis of Acupuncture Combined with Hyperbaric Oxygen Therapy in the Integrated Chinese and Western Medicine Treatment of Sudden Deafness. *Neural Injury and Functional Reconstruction* (2024) 19:644–647. doi: 10.16780/j.cnki.sjssgncj.20240207

22.	LIU G, GUO L, HE A, LU H, ZHAO W, Gao L, WANG Q, WANG Z, Longkang P, ZHANG R, et al. Clinical study on treatment of sudden hearing loss with combination of bloodletting and electroacupuncture. *Shaanxi Journal of Traditional Chinese Medicine* (2024) 45:980–984.

23.	Liu L, Cao Y, Wei K, Qiu Y, Wang H, Cui J, Huai D, Fei B. Clinical Application Value of Sphenopalatine Ganglion Acupuncture Combined with Vestibular Rehabilitation Training in Patients with Unilateral Sudden Deafness and Vertigo. *Chinese Archives of Otolaryngology-Head and Neck Surgery* (2025) 32:672–674. doi: 10.16066/j.1672-7002.2025.10.014

24.	LUO Q, XU Q, YE M, YANG X, XIAO H, HE F. Efficacy observation of along-meridian electroacupuncture combined with point injection for sudden hearing loss. *Shanghai Journal of Acupuncture and Moxibustion* (2024) 43:830–835. doi: 10.13460/j.issn.1005-0957.2024.08.0830

25.	MA X, ZHANG C, SUN D, SHANG G. Acupuncture Combined with Tongqiao Huoxue Decoction in the Treatment of Sudden Sensorineural Hearing Loss. *Guangming Journal of Chinese Medicine* (2024) 39:2859–2862.

26.	Nie Z, Shi X. Clinical Observation on the Treatment of Sudden Deafness with Acupuncture. *Guangming Journal of Chinese Medicine* (2023) 38:1740–1742.

27.	SUN L, DING D, PAN M, LI S, YU L, MA R. Clinical effect of Tiaoshen acupuncture in patients with refractory sudden deafness. *China Medical Herald* (2023) 20:138–142. doi: 10.20047/j.issn1673-7210.2023.25.30

28.	Wang T. Clinical Observation of Acupuncture Combined with Nasal Pinching and Air Puffing Method in the Treatment of Early-Onset Sudden Hearing Loss. *Journal of Practical Traditional Chinese Medicine* (2024) 40:2536–2537.

29.	Wang X. Effect of Warm-Tong Acupuncture on the Fengchi Acupoint in Treating Sudden Deafness and Its Impact on Hemorheology. *Inner Mongolia Journal of Traditional Chinese Medicine* (2025) 44:103–105. doi: 10.16040/j.cnki.cn15-1101.2025.10.035

30.	WANG Z, HUANG D. Clinical Study on Acupuncture with Method of Linggui Bafa Combined with Western Medicine for Flat Descent Sudden Deafness. *New Chinese Medicine* (2024) 56:111–115. doi: 10.13457/j.cnki.jncm.2024.20.021

31.	Xu H, Zhang R, Liu Y, Zeng X, Chen L. Clinical Observation on the Treatment of Sudden Deafness with Warm Acupuncture and Moxibustion. *Journal of Emergency in Traditional Chinese Medicine* (2024) 33:491–493.

32.	ZHANG H, CHEN L. Efficacy Analysis of Acupuncture Combined With Hyperbaric Oxygen in Adjuvant Treatment of Idiopathic Sudden Deafness. *Traditional Chinese Medicine Rehabilitation* (2023) 14:22–25. doi: 10.19787/j.issn.1008-1879.2023.08.007

33.	Zhang X, Cui J, Liu X. Observation on the Efficacy of Auricular Point Sticking Combined with Hyperbaric Oxygen Therapy for Sudden Deafness. *Journal of Practical Traditional Chinese Medicine* (2024) 40:1626–1628.

34.	ZHANG X, WANG Z, HUANG D. Clinical efficacy of batroxobin combined with acupuncture in the treatment of refractory sudden sensorineural hearing loss. *Guangdong Medical Journal* (2024) 45:1617–1620. doi: 10.13820/j.cnki.gdyx.20242151

35.	Zhang Y, Wei D, Jiang L, Zhu F, Liu W, Mei S. Clinical Observation on the Adjuvant Treatment of Idiopathic Sudden Deafness with Tinnitus Using Acupuncture at Four Points Around the Ear. *Journal of Practical Traditional Chinese Medicine* (2024) 40:2174–2176.

36.	ZHANG Y, TIAN C, LIU H. Observation on efficacy of transcutaneous electrical acupoint stimulation using a medium-frequency electrotherapy device combined with intratympanic injection of methylprednisolone for treatment of sudden sensorineural hearing loss. *Chinese Journal of New Clinical Medicine* (2025) 18:887–891.

37.	Zhang C, Liang J, Li Y, Wang X, Xing Y, Di X, Sun W, Bai P. Neck Seven-Line Method Combined with Periauricular Acupuncture for 33 Patients with Sudden Hearing Loss in Non-Acute Stage： A Randomized Controlled Trial. *Journal of Traditional Chinese Medicine* (2024) 65:1571–1577.

38.	HUANG X, QIN G, CHEN R, ZHOU W, PENG Q, LIANG Z, TU Y, LIN N, ZHOU S. Clinical effect of “Eryizhen” acupuncture combined with modified Conger Ximing Decoction in the treatment of refractory sudden deafness. *Guangxi Medical Journal* (2023) 45:550–552, 566.

39.	JIANG W, XIE X. 32 Cases of Acupuncture and Pushing Combined with Western Medicine Treating Sudden Deafness. *Zhejiang Journal of Traditional Chinese Medicine* (2022) 57:209–210. doi: 10.13633/j.cnki.zjtcm.2022.03.024

40.	LIU H, WANG L, PENG Q, JIA R. Effect of Acupuncture Combined with Tongqiao Huoxue Decoction on Hearing Level and Coagulation Function in Patients with Sudden Deafness. *Journal of Medical Information* (2022) 35:168–170.

41.	Wang F, Sun Y. Effects of Ear Acupoint Bean-Pressure Combined with Mindfulness Training on Negative Emotions and Sleep Quality in Patients with Sudden Deafness. *Journal of Clinical Nursing in Practice* (2022) 8:127–129.

42.	Wang G. Clinical Observation on the Treatment of Sensorineural Hearing Loss with Tongqi San Combined with Modified Tongqiao Huoxue Decoction and Auricular Acupuncture. *Guangxi Journal of Traditional Chinese Medicine* (2022) 45:5–7.

43.	WANG X, FAN F, LU Y. Clinical observation of tiaoshen kaiqiao acupuncture therapy in treating sudden deafness. *China’s Naturopathy* (2023) 31:53–56. doi: 10.19621/j.cnki.11-3555/r.2023.0117

44.	WEI C, SUN P. Clinical Observation on Tongdu Tiaoshen Acupuncture Combined with Hyperbaric Oxygen in the Treatment of Sudden Deafness. *Chinese Medicine Modern Distance Education of China* (2022) 20:120–121.

45.	Wu L, Cheng H. Observation on the Efficacy of Acupoint Plaster Application Combined with Auricular Acupoint Bean Pressure in Patients with Sudden Deafness. *Journal of Nursing and Rehabilitation* (2022) 21:51–53, 57.

46.	YU J, ZHU J, FENG W, DENG Q. Study on the Therapeutic Effect of Acupuncture on Sudden Deafness. *Zhejiang Journal of Traditional Chinese Medicine* (2022) 57:837–838. doi: 10.13633/j.cnki.zjtcm.2022.11.010

47.	ZHANG L, WANG Z, LI P. Observations on the Efficacy of Acupuncture with Eightfold Method of the Sacred Tortoise for Sudden Deafness and Its Effect on Hearing Levels and Hemorheology. *Shanghai Journal of Acupuncture and Moxibustion* (2022) 41:274–278. doi: 10.13460/j.issn.1005-0957.2022.03.0274

48.	Zhong Y, Wang B. The Therapeutic Effects of Chimai Acupoint Injection on the Sudden Deafness of Liver-fire Disturbing Upwards. *Chinese Medical Digest Otorhinolaryngology* (2022) 37:131–133. doi: 10.19617/j.issn1001-1307.2022.03.131

49.	DUAN H. Clinical observation of Fucong decoction combined with acupuncture at zhongzhu(TE 3) in treating sudden deafness. *China’s Naturopathy* (2020) 28:44–45. doi: 10.19621/j.cnki.11-3555/r.2020.0222

50.	FAN W, WU Y, CHEN Z, TANG Y, YAO M, ZHOU C, CUI X. Acupuncture in Combination with Hyperbaric Oxygen in Treatment of Low-Frequency Sudden Hearing Loss. *Chinese Journal of Otology* (2020) 18:98–102.

51.	HU M, LI G, CHEN W. Clinical effect of acupoint injection of dexamethasone and mouse nerve growth factor in the treatment of adolescent sudden deafness. *Clinical Research and Practice* (2020) 5:35–38. doi: 10.19347/j.cnki.2096-1413.202021013

52.	HU M, LI G, LI F. Clinical Study of Dexamethasone Combined with Mouse Nerve Growth Factor Acupoint Injection on Sudden Deafness in the Elderly. *Chinese Journal of Medicinal Guide* (2020) 22:565–569.

53.	Huang M. The Effect of Acupuncture on the Overall Response Rate in Sudden Deafness of the Qi Stagnation and Blood Stasis Type. *Journal of Jiujiang University(Natural Science Edition)* (2020) 35:95–97. doi: 10.19717/j.cnki.jjun.2020.03.025

54.	Jiang X, Shi X, Hu H, Zhang H, Han J, Wang X. Clinical Study on Treatment of Sudden Deafness with Traditional Chinese Medicine and Acupuncture. *Chinese Medical Digest Otorhinolaryngology* (2020) 35:361–362. doi: 10.19617/j.issn1001-1307.2020.05.361

55.	Liao C, Liang Q, Zhu L. Vitamin B12 Combined with Periaural Acupoint Injection for the Treatment of 34 Cases of Sudden Deafness. *Journal of External Therapy of Traditional Chinese Medicine* (2021) 30:44–45.

56.	LIAO H, HUA Q. Therapeutic Effect of Acupuncture Combined with Glucocorticoid on Sudden Deafness with Vertigo and Application of Vestibular Myogenic Evoked Potential in Diagnosis and Treatment. *Journal of Hubei University of Chinese Medicine* (2020) 22:99–102.

57.	Zhou X, Liao X, Huang J. Effect Analysis of Pressing Needle Therapy Combined with Peri-auricular Acupoint Injection in Treatment of Blood Stasis and Ear Orifice Type of Sudden Deafness with Sleep Disturbance. *Chinese Community Doctors* (2022) 38:65–67.

58.	SONG H, XU F, CHEN Y, XIONG D, LIN Y, LIU L, TAN R, ZHAO X, ZHONG Q, CAI Y. Effect of Acupuncture Combined with Method of Expelling Phlegm and Removing Blood Stasis and Soothing Liver for Resuscitation on SAS Score in Patients with Refractory Sudden Sensorineural Hearing Loss. *Liaoning Journal of Traditional Chinese Medicine* (2021) 48:176–180. doi: 10.13192/j.issn.1000-1719.2021.02.047

59.	Sun J. The short and long-term effects of acupoint acupuncture plus western medicine on sudden deafness. *Clinical Journal of Chinese Medicine* (2021) 13:27–29.

60.	WANG X, ZHENG S, FANG F, LIN B. Clinical Effect of Acupuncture at Three Points of the Neck Treating Sudden Deafness and Its Influence on Hemorheology. *Zhejiang Journal of Traditional Chinese Medicine* (2021) 56:213–214. doi: 10.13633/j.cnki.zjtcm.2021.03.041

61.	YE Z, LI J, TONG Y, LUO S, MA J. The Clinical Observation of Hyperbaric Oxygen Combined with Acupuncture in the Treatment of Sudden Deafness. *Chinese Medicine Modern Distance Education of China* (2021) 19:91–94.

62.	Yu Z, Sun H. Effect of methylprednisolone sodium succinate for injection at auricular points on patients with low frequency sudden hearing loss. *China Medicine* (2021) 16:913–916.

63.	Zhang X, An J, Ren Y, Huang L, Ma A. The Effect of Fang’s Scalp Acupuncture Combined with Acupoint Injection on the Treatment of Sudden Deafness of the Qi Stagnation and Blood Stasis Type. *Henan Medical Research* (2020) 29:6479–6481.

64.	ZHOU H, QI P, ZHAO Y. Clinical Observation of Applying Kaiqiao Fucong Acupuncture in the Treatment of Sudden Deafness ( Liver-fire Exuberance Syndrome). *Journal of Sichuan of Traditional Chinese Medicine* (2021) 39:198–202.

65.	Zhou S. The Effect of Traditional Chinese Medicine Acupuncture on Hemorheology in Patients with Sudden Deafness. *Practical Clinical Journal of Integrated Traditional Chinese and Western Medicine* (2020) 20:55–56, 124. doi: 10.13638/j.issn.1671-4040.2020.13.026

66.	Gao Y, Han N, Li G. Effect of postauricular acupoint injection of methylprednisolone sodium succinate combined with modified Longdan Xiegan decoction in treatment of sensorineural hearing loss. *International Medicine and Health Guidance News* (2025) 31:2439–2443. doi: 10.3760/cma.j.cn441417-20250221-14030

67.	Yu C, Yu F, Deng H, Li J, Huang C, Li H. Clinical Efficacy of Electroacupuncture and Acupoint Injection in the Treatment of Sudden Deafness. *International Medicine and Health Guidance News* (2022) 28:2522–2527. doi: 10.3760/cma.j.issn.1007-1245.2022.18.002

68.	Yang J, Li X, Li X, Huang Y, Wang X, Bai P, Liu J, Zhou W. Clinical Study on Acupuncture Combined with Hyperbaric Oxygen and Medication in the Treatment of Sudden Deafness. *Chinese Journal of Nautical Medicine and Hyperbaric Medicine* (2022) 29:644-646,651.

69.	Huang D, Zhang X, Zhang Y, Gu Y. Auricular point pressing combined with community health education for patients with sudden deafness. *International Medicine and Health Guidance News* (2021) 27:1653–1655. doi: 10.3760/cma.j.issn.1007-1245.2021.11.018

70.	Zhang Y, Huang D, Gu Y, Zhang X. Auricular point pressing combined with progressive muscle relaxation training for patients with sudden deafness. *International Medicine and Health Guidance News* (2021) 27:1351–1353. doi: 10.3760/cma.j.issn.1007-1245.2021.09.020
